# Clinician and policymaker perspectives on the barriers and enablers to implementing and scaling up integrated postpartum intrauterine contraceptive services within maternity care in Nepal: a qualitative study

**DOI:** 10.1016/j.lansea.2025.100599

**Published:** 2025-05-14

**Authors:** Pramila Rai, Denise A. O'Connor, Ilana N. Ackerman, Ganesh Dangal, Surya Prasad Rimal, Pabitra Rai, Rachelle Buchbinder

**Affiliations:** a School of Public Health and Preventive Medicine, Monash University, Melbourne, Australia; b Kathmandu Model Hospital, Kathmandu, Nepal; c B. P. Koirala Institute of Health Sciences, Sunsari, Nepal; d Faculty of Health, Deakin University, Melbourne, Australia

**Keywords:** Postpartum contraceptives, Postpartum intrauterine contraceptive devices, PPIUCD, PPIUD, Family planning, Barriers, Enablers, Qualitative study, Nepal

## Abstract

**Background:**

Integrating postpartum family planning services within maternity care, specifically counselling about family planning and postpartum intrauterine contraceptive device (PPIUCD) insertion, may help reduce unintended pregnancies and related complications. This study explored factors affecting the implementation and scale-up of integration of these services within maternity care in Nepal from the perspectives of healthcare providers and policymakers.

**Methods:**

For this qualitative study, we conducted in-depth, semi-structured interviews remotely with healthcare providers and policymakers across all seven provinces of the Nepali maternal healthcare sector until theme saturation was achieved. Potentially eligible participants were invited through publicly available e-mail addresses, personal contacts, snowball sampling, and social media advertisements. Respondents were assessed for eligibility and subsequently recruited. The Consolidated Framework for Implementation Research and Theoretical Domains Framework guided our inquiry and analysis. We transcribed the interviews verbatim, translated the transcripts into English and analysed them using thematic analysis.

**Findings:**

Based on 26 interviews, we identified five major barrier themes relating to care recipients, healthcare providers, health facilities and the health system. The themes included: (i) the perceived inadequate awareness and low desire for PPIUCDs among care recipients, (ii) PPIUCD-specific issues, (iii) inadequate capacity and capability to deliver the services, (iv) inadequate investment and priority, and (v) contextual factors such as pelvic inflammatory diseases and hygiene considerations. Some contrasting views were reported between healthcare providers and policy participants. Policy participants emphasised insufficient efforts by healthcare providers to provide counselling and PPIUCD, while healthcare providers identified PPIUCD-related issues (e.g., complexity of the insertion procedure) as a significant barrier hindering their efforts. Both healthcare providers and policy participants identified inadequate investment in and priority on integrating postpartum contraceptive services, including PPIUCD, as another important factor. Participants indicated that there is an urgent need to implement effective integrated counselling and contraception services.

**Interpretation:**

Greater investment is needed to address multilevel barriers to implementing and scaling up integrated postpartum family planning services, particularly PPIUCD insertion within maternity services in Nepal. Priority should be given to health education for care recipients and the community, capacity building (ensuring care providers are capable), and upgrading of health facilities.

**Funding:**

Monash International Postgraduate Research Scholarship and Monash Graduate Scholarship.

**Research in context**

**Evidence before this study**

To understand the barriers and enablers to integrating counselling for postpartum family planning and postpartum intrauterine contraceptive device (PPIUCD) insertion in maternity care in Nepal, we conducted an extensive literature search from inception to January 2024 using the keywords “PPIUCD,” OR “PPIUD,” OR “postpartum intrauterine contraceptive devices,” OR “intrauterine devices,” OR “postpartum intrauterine devices,” OR “Postpartum contraceptives, “OR “Postpartum family planning,” AND “Nepal” across databases such as PubMed, Google Scholar, Scopus, and Nepal Journals Online (NepJOL) with no filter for publication year. The search yielded 514 results, of which 75 records were relevant to this topic and setting. Fear of side effects, complications, inadequate counselling, misconceptions and social influences were identified as barriers to the uptake of PPIUCD from the perspectives of care recipients. Work overload, lack of private space for counselling, scarcity of family planning devices, and insufficient support from hospital management were identified as barriers from the perspectives of healthcare providers. We did not identify any studies that had explored system-level barriers or enablers to the integration of counselling for postpartum family planning and PPIUCD insertion in maternity care from the policymaker perspective.

**Added value of this study**

Building upon earlier studies that primarily focused on Nepali care recipients’ opinions and perspectives, this study explored the barriers and enablers through the lens of healthcare providers and policymakers in Nepal. To minimise the risk of overlooking important factors, the inquiry was guided by established theoretical frameworks, which encompass various implementation domains such as policy level, organisation level, care recipient and provider level, and counselling and PPIUCD-related factors. This study not only enhances understanding of what is important to providers and policymakers but also highlights the differences in perspectives between them.

**Implications of all the available evidence**

Our study confirms that greater investment is urgently needed to address multilevel barriers to the implementation and scale-up of integrated postpartum family planning services within maternity services in Nepal. Significant barriers include the low perceived demand for postpartum family planning counselling and PPIUCD insertion among care recipients, and limited health care system and provider capacity and capability to deliver these services. Based on the current study findings, an appropriate multi-level programme can be developed to increase awareness of PPIUCD, improve health service capacity and capability, and increase system-level investment for integrating this service.

## Introduction

Family planning, a pillar of the international Safe Motherhood initiative, is a cost-effective measure for lowering maternal and infant mortality and morbidity, reducing abortion rates, and empowering women.^1^, ^2^, ^3^ It lowers the risk of maternal death by preventing unwanted and high-risk pregnancies.^4^^,^^5^ In Nepal, the National Family Planning Costed Implementation Plan for 2015–2020 estimated there would be 230 fewer maternal deaths per year by 2030 with a broader family planning programme.^6^

Despite several expected benefits,^6^^,^^7^ the uptake of family planning strategies in Nepal has been suboptimal.^8^^,^^9^ Between 2015 and 2019, it was estimated that there were 60 unwanted pregnancies per 1000 reproductive-aged women,^10^ with approximately two-thirds ending in abortion.^8^ Although there has been a slight increase in the use of traditional methods of contraception such as withdrawal and rhythm (7% in 2011 to 15% in 2022), the use of modern contraceptives such as male and female sterilisation, intrauterine contraceptive device (IUCD), injectables, condom, pill, implants has stagnated at 43% since 2011, and the unmet need of family planning for birth spacing or limiting childbirth is 20.8%, highlighting poor uptake of family planning services.^9^

Nepal aims to achieve its Sustainable Development Goal of increasing the contraceptive prevalence rate to 75% by 2030.^11^ Integrating family planning services within maternity care is one promising strategy to achieve this, given the low rate of postpartum contraceptive use in Nepal.^12^ Only 26% and 40% of women (or their partners) use modern postpartum contraceptives at six months and one year postpartum respectively, comparable to India (29%, 37%) but considerably lower than in neighbouring Bangladesh (56%, 70%).^13^, ^14^, ^15^ Additionally, less than half (∼43%) of women in Nepal exclusively breastfeed for six months, leading to a shorter duration of lactational amenorrhea^16^ and 20% have a birth spacing of less than 24 months.^9^

While counselling for postpartum family planning is recommended in Nepali maternity services, its implementation varies widely. For example, further analysis of the Nepal Health Facility Survey 2021 indicated that only 1% of women were observed receiving counselling on family planning methods during antenatal care.^17^ Nepal Demographic and Health Survey 2022 reported that only 25.3% of postpartum mothers reported discussing family planning postpartum.^9^ Effective postpartum family planning and effective postpartum contraception may prevent unintended pregnancies as well as closely spaced pregnancies in the first 12 months following childbirth.^12^

An option for effective and safe contraception postpartum include postpartum intrauterine contraceptive devices (PPIUCDs) insertion immediately after childbirth, which has only recently been explored in the Nepali health context.^18^ PPIUCDs can be safely inserted within 10 min of placenta delivery or in the first 48 h of delivery.^19^ After 48 h, insertion should be delayed for 4–6 weeks to decrease the chance of infection.^19^, ^20^, ^21^ The advantage of PPIUCDs over subdermal implants is that they can be inserted immediately after childbirth without needing an extra procedure and their advantage over progestin-only injectables is that there is no need to wait six weeks.^19^ PPIUCDs are safe for breastfeeding women and do not require frequent visits to health facilities (in contrast to short-acting hormonal contraceptives including injectables), making them a viable choice in settings where physical access to healthcare is limited.^21^ PPIUCDs are safely used for postnatal women around the world^22^^,^^23^ and the Nepalese National Clinical Standard for Reproductive Health also recommends PPIUCD insertion immediately after birth as an appropriate method of contraception.^20^

PPIUCD integration with maternity services is cost-effective in low-income countries such as Bangladesh and Tanzania^24^ and effective in increasing contraceptive uptake in several settings, including Nepal.^18^^,^^25^^,^^26^ However, its implementation in practice is a multifaceted process where multiple organisational, individual, financial, and sociocultural factors interact.^27^

To optimise implementation and scale up of postpartum family planning services including counselling and PPIUCD within routine maternal care requires a nuanced understanding of these factors. While previous studies have explored the perspectives of care recipients,^28^, ^29^, ^30^ only one study to date has investigated the perspectives of healthcare providers,^31^ and none have explored broader health policy maker perspectives. We undertook this study to identify barriers and enablers to the implementation and scale-up of integrated postpartum family planning services, specifically family planning counselling and PPIUCD insertion soon after birth, within existing maternity care services in Nepal, from the perspectives of healthcare providers and policy makers.

## Methods

### Study design and setting

This qualitative study involved relevant healthcare professionals (obstetricians, medical officers, nurses, midwives and auxiliary nurse midwives), healthcare administrators, and policymakers in Nepal. It reported key aspects of the research process according to the Consolidated Criteria for Reporting Qualitative Research (COREQ) guidelines.^32^ We obtained ethical approval from the Monash University Human Research Ethics Committee (Project ID 34471, approval date: Feb 24, 2023) and the Nepal Health Research Council (Ref. No. 2376, approval date: March 19, 2023). The Department of Health Services, Nepal, also provided administrative approval for this research.

### Theoretical frameworks

**Fig. 1:**
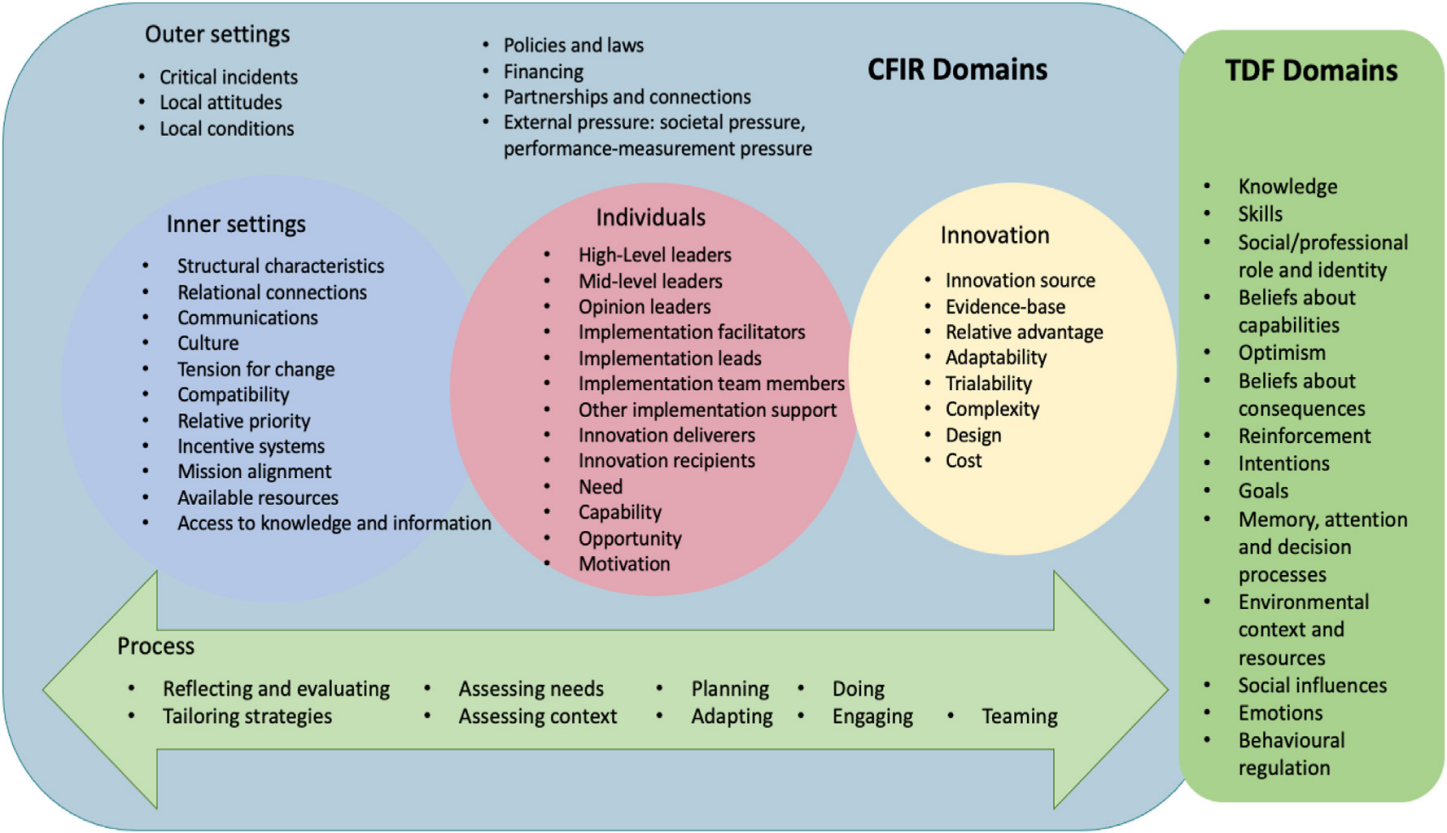
**Overview of Consolidated Framework of Implementation Research (CFIR) and Theoretical Domains Framework (TDF) domains**.

Two well-established theoretical frameworks that provide complementary guidance, the Consolidated Framework for Implementation Research (CFIR) and the Theoretical Domains Framework (TDF), guided our inquiry.^33^, ^34^, ^35^ The CFIR framework, which includes high-level categorical domains such as outer setting (e.g., policy, laws), inner setting (e.g., organisation), innovation (operationalised in this study as postpartum family planning counselling and PPIUCD insertion) (Fig. 1) that are relevant in any implementation phase from planning to evaluation, guided the areas to explore during PPFP implementation.^36^, ^37^, ^38^

We further incorporated the TDF to enable a more detailed understanding of individual-level factors that could influence implementation and scale-up (Fig. 1), in addition to the broader organisational-level factors more elaborated within the CFIR. The frameworks we used also emphasised the importance of including participants that could provide perspectives about influencing factors relating to different domains (health facilities, policy settings). For instance, the outer domain of the CFIR and the environmental context and resources domain from the TDF suggest that we look at perspectives beyond health facilities. Supplementary Tables S1 and S2 provide further detail on how the CFIR and TDF domains were considered in the context of this study.

### Participants

Following the Action, Actor, Context, Target, Time (AACTT) framework, we identified the stakeholders with an active role in delivering, ensuring, institutionalising and advocating the implementation of integrated PPFP counselling and PPIUCD integration services (Supplementary Table S3).^39^ For example, in the implementation of this PPFP service, the healthcare providers (Actors) need to provide family planning counselling from the first contact with maternity care and provision of the PPIUCD service (Action), while policymakers (Actors) contribute to advocacy, funding, planning (Actions) for implementation. Therefore, policymakers, for example, could provide a deeper understanding of the outer setting (e.g., policies, laws, local conditions, broader attitudes), whereas healthcare providers are expected to provide perspectives of greater relevance to practices in healthcare delivery settings. Hence, we included two participant streams in this study.

The first stream included healthcare providers directly involved in delivering maternity care, with at least six months of experience (including obstetricians, medical officers, nurses, midwives, and auxiliary nurse midwives). A second policy stream included professionals working as health system managers or policymakers at the Department of Health, as well as professionals involved in advocacy or guiding and/or directing the delivery of maternity services either in health facilities or in the health system more broadly. Potentially eligible individuals who could not participate in a remotely conducted interview via audio/video conference or telephone were ineligible for logistic reasons.

### Recruitment and method of approach

Various channels were utilised to advertise the study to potential participants. Study invitations were sent to a publicly available list of doctors and nurses, health system managers, and policymakers through the relevant professional body or by contacting the contact person named on the website of the major professional bodies such as the Nepal Society of Obstetricians and Gynaecologist (NESOG), Nursing Association of Nepal (NAN), or health facilities.

**Fig. 2:**
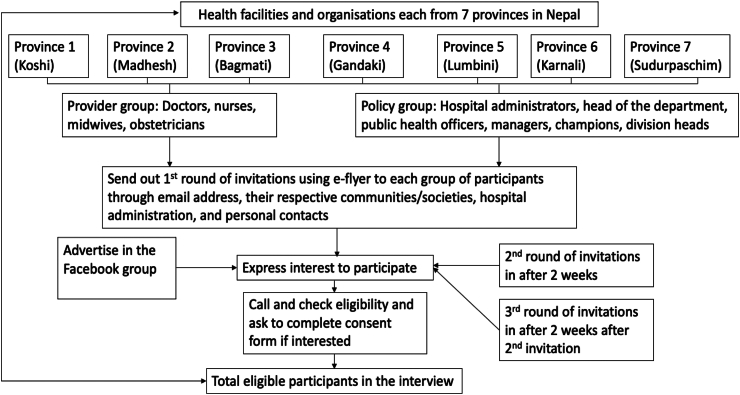
**The recruitment process for the research**.

The study was also advertised via the Facebook group of public health professionals in Nepal, and snowball sampling was used to identify other potential participants. We employed a maximum variation sampling approach,^40^ selecting participants with different roles and qualifications from different levels of health institutions across all seven provinces of Nepal to capture diverse experiences and perspectives on the factors influencing implementation. Individuals meeting the eligibility criteria were invited to take part in an interview. Once they expressed their intention to participate in the study, an explanatory statement and consent form were provided to eligible participants. The participants provided written informed consent before or during the interview by returning the signed consent form through email or directly signing it using Google Forms. The recruitment process is outlined in Fig. 2.

### Sample size

We initially aimed to include 12 healthcare providers and 12 policymakers, unless new themes continued to emerge, following the principle of ‘data saturation’ or ‘information redundancy’.^41^, ^42^, ^43^ Using a pragmatic approach, we ceased data collection once information started to be recurrent.^41^^,^^42^ We treated policy participants and healthcare providers as separate groups based on heterogeneity, and theme saturation was considered and observed separately for these groups.

### Data collection

We (PR (1), DAOC, INA, SPR, and RB) developed a semi-structured interview guide (Supplementary Information) for healthcare providers and policy participants informed by the CFIR and TDF domains and previous literature. The interview schedule was translated into Nepali by the first author (Supplementary Information). The interview schedule was piloted in two interviews, and minor changes were made to improve the clarity and flow of questions. As the questions were open-ended and non-leading, verbal prompts were used during the interviews, where needed, to increase understanding.

The interviews commenced in April 2023, and the last interview was conducted in December 2023. All interviews were conducted by the first author PR (1), a female PhD candidate with a Bachelor of Science in Nursing and a Master of Public Health who is trained in qualitative research and is experienced in interviewing. Some of the participants were personal or professional contacts of the first author PR (1), given her prior professional experience working in the Nepal health system.

Interviews were conducted in the Nepali language (with occasional use of English) via Zoom, Viber, WhatsApp, and Messenger, according to participant preferences. The interviewer remained objective by giving interviewees ample time to elaborate on their responses without reacting, focusing on the issues discussed, and using probing questions when necessary. The interviews took on average, 40–60 min per participant. There were no incomplete interviews. All interviews were electronically recorded. The recordings were transcribed verbatim in Nepali and translated into English by PR (1) and SPR (both proficient in English and Nepali). We returned 15% of the transcripts to the participants for review and checking of accuracy. All transcripts were imported into NVivo qualitative data analysis software 14.23.0 (Lumivero) for coding and analysis.

### Data analysis

We conducted a thematic analysis of interview data,^44^ beginning with inductive coding of the transcripts. Initially, codes were generated based on the information derived from the data (inductive coding). Similar codes were subsequently organised to develop overarching themes and identified subthemes, where applicable, facilitating a comprehensive understanding of the data. We next mapped the identified themes to the CFIR and TDF domains. PR (1) was responsible for generating initial codes and themes. PR (1) and PR (2) independently coded 20% of the interview transcripts to reach consensus. PR (1), DAOC, INA, and RB were involved in generating themes and subthemes, selecting and finalising exemplar quotes, and mapping to the two theoretical frameworks. Any disagreements in the coding, themes, and subthemes were resolved through discussion with the research team. Where illustrative quotes are provided in this paper, these are reported verbatim (after translation into English). The data analysis steps, and the role of participants are provided in detail in the Supplementary Figure S1.

### Role of the funding source

The funder of the study had no role in study design, data collection, data analysis, data interpretation, writing of the report or the decision to submit paper for publication.

## Results

We interviewed 26 participants, including 14 healthcare providers and 12 policy professionals. During the interview process, we started to observe recurrent themes after 10 interviews with healthcare providers and stopped recruitment after an additional four interviews, given recurring themes evident from participants’ responses to the interview questions. Similarly, we stopped interviewing policy participants after 12 interviews as recurring themes were evident.

An overview of participant characteristics is provided in Table 1. The healthcare provider participants had an average age of 37.7 years, while the policy participants had an average age of 47.7 years. The healthcare providers had an average work experience of 13.4 years (range 3–23 years). The policy participants had an average work experience of 24 years (range 14–39 years). All the provinces had at least one healthcare provider representative, and policy participants represented local, provincial or central-level organisations.

**Table 1:** Demographic characteristics of study participants.

Participants	Profession or position	Province or representation	Organisation	Gender
HCP1	Doctor	Sudurpaschim	Local Hospital	Female
HCP2	Doctor or Medical officer	Karnali	District hospital	Male
HCP3	Nurse or Auxiliary nurse midwife	Sudurpaschim	District hospital	Female
HCP4	Nurse or Nurse in charge	Karnali	District hospital	Female
HCP5	Nurse or Staff Nurse	Koshi	Tertiary hospital	Female
HCP6	Nurse or Senior Nursing administrator	Madhesh	Tertiary hospital	Female
HCP7	Nurse	Bagmati	Municipality hospital	Female
HCP8	Doctor (OB/GYN)	Bagmati	District hospital	Female
HCP9	Nurse	Gandaki	Tertiary hospital	Female
HCP10	Doctor or Medical officer	Madhesh	Tertiary level hospital	Female
HCP11	Doctor (OB/GYN)/Assistant Professor	Gandaki	Tertiary level hospital	Male
HCP12	Doctor (OB/GYN)/Assistant Professor	Koshi	Tertiary/teaching hospital	Male
HCP13	Nurse or Auxiliary nurse midwife	Koshi	Health Post	Male
HCP14	Doctor (OB/GYN)	Lumbini	Provincial hospital	Female
PP1	Public health officer	Local	Health office, metropolitan city	Male
PP2	Senior Public health officer	Provincial	District health office	Male
PP3	Senior Public health officer	Local	Health division in municipality	Male
PP4	Family planning inspector	Local	Health division in municipality	Male
PP5	Senior branch manager	Local	NGO	Male
PP6	Board member, co-founder, advisor	Central	National committee, professional society	Female
PP7	Country director	Central	NGO	Male
PP8	President	Central	Professional society	Female
PP9	MNCH advisor (OB/GYN)	Central	Private hospital and NGO	Female
PP10	Nursing administrator	Central	Central government office	Female
PP11	Program director	Central	NGO	Female
PP12	Division director	Central	Central government office	Male

Note: OB/GYN, obstetrician and gynaecologist; PP represents policy stream, and HCP represents the healthcare providers; NGO, Non-governmental organisation; MNCH, maternal, neonatal and child health.

### Perceived barriers to the uptake of the integrated postpartum family planning and PPIUCD service

From our interviews with health providers and policymakers, we identified five key barrier themes relating to care recipients, healthcare providers, health facilities, the innovation (counselling for postpartum family planning and PPIUCD insertion), and the health system. Table 2 presents the list of codes that contributed to the barrier subthemes and themes, mapped to the domains and constructs of the theoretical frameworks. Selected illustrative quotes for these barrier themes are provided in Table 3 and the complete list of quotes for each theme is available in the Supplementary Table S4.

**Table 2:** Identified codes, barrier themes, subthemes, and mapping to theoretical frameworks.

Codes	Themes	Subthemes	TDF domains	CFIR domains or constructs
1 Unaware about the PPIUCD option, safety and benefits 2 No coverage in media 3 Inadequate awareness about fertility, conception post pregnancy	Recipients' limited awareness of and desire for PPIUCD as perceived by healthcare providers	Limited awareness	Beliefs about consequences; Intentions; Social influences; Knowledge; Emotion	Outer setting domain: Local attitudes Inner setting domain: Relative priority Individuals domain: Recipients' motivation and capability, need
4 Misleading information about returns of fertility 5 Misleading information about the need of contraceptive 6 Inadequate or misleading information about the PPIUCD 7 Myths attached to IUCD		Misconceptions		
8 Generalised fear relating to complications 9 Not taking ownership of health 10 Insensitive reproductive health behaviour and shyness 11 Contraceptive use not taken as tool to satisfied sexual life 12 Negativity attached to the sexual health 13 Women's issue or no male involvement		Attitudes		
14 Some religions prohibit use of contraceptives or IUCD 15 Women seek consultation and permission for contraceptive use 16 Women prefer what contraceptives their peers and neighbours are using 17 Unaware and unsupportive family		Social and cultural influences		
18 PPIUCD insertion procedure is not that easy 19 Training and supervision are required for insertion of IUCD 20 Dealing with complication is complex	Postpartum intrauterine contraceptive device issues, preferences and competitive interest	Complexity of PPIUCD procedure	Skills, Intentions, Beliefs about capabilities, Beliefs about consequences, Emotion	Innovation domain: Innovation complexity; Innovation design Individuals domain: Deliverers' motivation and capability, need; Recipients' motivation and capability, need
21 There are occurrences of rare complications 22 Rare complications can be dangerous 23 There are side effects as well		Health conditions, side effects and complications		
24 Women prefer implant for long-acting contraceptive 25 Women are aware of and prefer Depo-Provera 26 There are long-time users of hormonal contraceptives		Preferred alternatives		
27 May have to deal with complications 28 Healthcare provider was accused of negligence 29 Extra responsibility to manage service of patients		Beliefs about consequences		
30 Delivering PPIUCD is not urgent 31 Dealing childbirth complication is more important 32 Not providing PPIUCD would not take women's life		More urgent care takes priority		
33 Low doctor patient ratio 34 maternity service is busy 35 High workload at OPD and ward 36 Turnover of trained staff and lack of permanent employee	Inadequate capacity and capability (Health resource management)	Inadequate staff and high workload	Skills; Beliefs about capabilities; Environmental context and resources; Social or professional role and identity	Individuals domain: Deliverers' motivation, opportunity and capability Inner setting domain: Available resources; Work infrastructure
37 There are untrained staff 38 There is a need of training for staff 39 Lack of midwives		Inadequate training		
40 Not adequate counsellors 41 Lack of counselling training to providers 42 No adequate time to counsel 43 Selective counselling with counselling of only vulnerable patients		Variability in family planning counselling practice		
44 Less priority placed by obstetricians 45 Lack of do-it attitudes 46 All providers are not equally dedicated 47 Providers may be biased due to poor hygiene 48 Inconvenient procedure for integration 49 Shift in professional interest (towards advanced procedure)		Attitudes of healthcare providers		
50 No separate counselling rooms and lack of counsellors 51 Inadequate staff and high workload 52 Inadequate training 53 Lack of ultrasound machine 54 Not all health facilities are capable of providing service 55 Inadequate physical infrastructure of health facilities	Inadequate investment and priority	Limited resourcing and/or preparedness of health facilities	Reinforcement; Goals; Environmental context and resources; Behavioural regulation; Emotion	Individuals domain: Implementation leaders' opportunity and motivation; Implementation support; Critical incidents; Deliverers' need, motivation, opportunity and capability Inner setting: Incentive systems; Tension for change; Funding; Available resources; Space Outer setting: Critical incidents; Partnerships and connections; Financing
56 Lack of supervision/supervisors for postpartum family planning 57 Lack of review and monitoring 58 Don't know who is the one for monitoring 59 Understanding of health among executives 60 Negotiation with elected executives		Issues with oversight		
61 The patients bear cost when dealing complications 62 Additional cost of the ultrasound procedure, travel 63 Overall less funding in health and funding in family planning is minimal 64 Funding crisis or reduction due to less interest of supporting external partner 65 Dependency on external partners 66 Pandemics, war also affect the funding situation		Cost and funding		
67 No target set for postpartum family planning service 68 No demand from the recipients		Little appetite or compulsion or obligation for change		
69 Minimal incentive 70 No incentive 71 Belief that incentive can motivate implementation		Incentive provision		
72 Long–distance relationship due to migrant working condition 73 Endemic infection status of pelvic inflammatory diseases 74 Vaginal discharge 75 High prevalence of HIV/AIDS in certain regions 76 Underutilization of basic facilities	Contextual factors	Spouse, hygiene considerations and endemic infections	Environmental context and resources; Emotion	Outer setting: Local conditions; Critical incidents

Note: CFIR, Consolidated Framework for Implementation Research; IUCD, Intrauterine Contraceptive Device; OPD, Outpatient department; PPIUCD, Postpartum Intrauterine Contraceptive Device; TDF, Theoretical Domains Framework.

**Table 3:** Examples of illustrative quotes for barrier themes.

Barrier themes	Illustrative quotes
Recipients' limited awareness of and desire for PPIUCD as perceived by health	“We have also perceived a knowledge gap. We have encountered women who had conceived when the last child was still under a year old. They have a conception that they don't need to use any contraceptives if their menstruation is not returned.” -PP7, Director, Central level “There are rumours about it getting lost once placed in the uterus. They have a misconception that IUCD causes cancer.” -HCP3, Nurse, Sudurpaschim province “After counselling, some patients wanted to use it, but some used to raise concerns of what they heard about complications such as losing of thread, displacement to the intestine.” -HCP10, Doctor, Madhesh province “Some people also complained of contraceptive failure as husbands threw the IUCD out during the intercourse. Such things are not even reported in the research.” -HCP8, Doctor, Bagmati province
PPIUCD issues, preferences, and competitive interest	“Insertion of PPIUCD is a complex procedure and not all healthcare providers are trained for PPIUCD. It needs trained persons to insert and remove this contraceptive. The easiest are pills and Depo-Provera.” -HCP2, Medical officer, Karnali province “I personally like it. However, it is very difficult to get patients' acceptance for this contraceptive. Implant is preferred among postpartum family planning contraceptives.” -HCP7, Nurse, Bagmati province
Inadequate capacity and capability	“What we don't have is adequate human resources, time and a system to motivate them.” HCP9, Nurse, Lumbini province “Tertiary level hospitals like ours are being overused.” -HCP14, Doctor, Lumbini province “Our 8 facilities don't have all these services and it was because of shortage of trained human resources.” -PP4, Family planning inspector, Bagmati province “The community health workers, such as female community health volunteers, are also almost non-functional in urban and peri-urban areas. They are actively working only in remote areas.” -PP12, Director, Central level “I won't say we can counsel every woman in the outpatient department during antenatal visits as we see about 200–300 recipients in a day, so I won't say we can counsel everyone, but at least we can counsel 10 of them with important indications such as gravida more than 3.” -HCP10, Doctor, Madhesh province “When we don't have enough resources, we can't expect them to provide quality counselling. Be it [tertiary hospital] or [central hospital], or any big hospitals with high obstetric caseloads, they already have a resource crunch” -PP12, Director, Central level
Inadequate investment and priority	“Counselling is impractical once they start having labour pain. So, it is better to have a separate dedicated counsellor. Some budget can be allocated for this.” -HCP9, Nurse, Lumbini province “But as family planning is not directly related to mortality, that's why it doesn't have special review programmes.” -HCP6, Nurse, Madhesh province “But the main setback is the loss of copper-T thread, and when they don't feel or see the IUCD thread, the recipients are required to pay around 3–4 thousand for an ultrasound procedure to find if the copper-T was dislodged and for its removal. … … Initially, it seemed free to patients for insertion, but they perceived it as not free when they faced complications. Because of that, the patients gradually started to tell one another, and consequently, people were reluctant.” -HCP12, Doctor, Koshi province “Practically, family planning is an old program; more emphasis is placed on the new programs. New projects are created for new issues. Due to the fact that there are currently fewer projects in family planning, the level of implementation and service delivery has been overshadowed in some places.” -PP5, Sr. Branch manager, Koshi province “What's more, there is a national trend of investing less in the health sector among other ministries. Because of that, even the province level also invests less in health sector. And at the local level, budget is mostly allocated for building roads and other infrastructures rather than the health sector.” -PP10, Nursing administrator, Central level “To say the truth, postpartum family planning counselling doesn't get enough priority.” -HCP9, Nurse, Lumbini province “We don't have any target such as PPIUCD prevalence rate to achieve” -HCP1, Doctor, Sudurpaschim province
Contextual factors	“And about 50% of the recipients report that their husbands reside somewhere else due to job. We do our best to provide comprehensive counselling, but mostly they don't want to use it as they reasoned that their husbands are abroad.” -HCP9, Nurse, Lumbini province “It's a good option, but there are hygiene issues in this locality/district. Women sleep outside in a hut when menstruating, and don't wear panties and pads, so there is a high risk of infection. Due to this, there is rarely a woman who doesn't suffer from pelvic inflammatory disease.” -HCP4, Nurse, Karnali province “In fact, we also didn't recommend in the past using IUCD due to the high prevalence of sexually transmitted infections and HIV in this region. As most men were migrant workers in India, the HIV prevalence was very high, and we especially advocated for the use of barrier methods.” -PP2, Sr. Public Health Officer, Sudurpaschim province

Note: IUCD, Intrauterine Contraceptive Device; PPIUCD, Postpartum Intrauterine Contraceptive Device.

#### Theme 1: recipients’ limited awareness of and desire for PPIUCD as perceived by health professionals

Participants expressed concerns about recipients' limited desire for PPIUCD due to low awareness, misconceptions, negative attitudes and social and cultural influences leading to perceived low demand for the service.

##### Low awareness

Participants noted that many women were unaware of PPIUCD use, preventing them from seeking the service. They expressed that although other contraceptives (especially short-acting contraceptives such as Depo-Provera [medroxyprogesterone acetate] and condoms) are advertised, PPIUCD was not promoted well in Nepal, implying a need for more information provision from healthcare providers.

“We need to advertise all available postpartum family planning services, especially PPIUCD. Just as there is routine about when to give iron and calcium tablets during pregnancy, family planning component should also be added to the regular antenatal care.”

HCP7, Nurse, Bagmati province

##### Misconceptions

In their experiences, participants reported that women and families had misconceptions about PPIUCDs. They noted that inadequate and misleading information regarding side effects and severe complications induced fear. Many misconceptions about PPIUCDs were reported by the providers, such as *“PPIUCD reaching to heart”, “causing cancer”*, and “*piercing the uterus and reaching internal organs*”. In addition, they stated that women declined PPIUCD insertion as they falsely believed that family planning was not needed until the return of menstruation or at least two years after childbirth.

##### Attitudes

Participants highlighted negative attitudes towards PPIUCD use among women due to current misconceptions. They also noted that women avoided using PPIUCDs due to privacy concerns during the insertion procedure, which deterred them from choosing it as their preferred contraceptive method. The participants also noted fears among women relating to the occasional complications of IUCDs.

“They believe that family planning is only necessary when they resume menstruation. That is why patient acceptance is very low.”

HCP8, Doctor, Bagmati province

##### Social and cultural influences

Although participants acknowledged that contraceptive decision-making should be based on the woman's preference and consideration of how her body reacts, they described how a woman's decision was also affected by the people around her. The participants stated that there was a lack of autonomy in women's decision-making as contraceptive use was highly influenced by their partners, family members, peers, and cultural values. While there were occasions when women declined to use PPIUCD due to objections from their husbands, in-laws and family members, it was considered important for women to align with what their peers were using. The participants highlighted that the lack of awareness from family members about postpartum family planning methods, including PPIUCDs, is a significant barrier for women.

“Family planning is still important, but women do not use family planning due to reasons such as family limitations, lack of support from husband and family support, and insufficient knowledge about family planning.”

PP3, Sr. Public Health Officer, Local level

#### Theme 2: postpartum intrauterine contraceptive device issues, preferences and competitive interest

Notably, this theme emerged primarily from the healthcare provider interviews.

##### Complexity of PPIUCD procedure

Healthcare providers indicated that they needed to be skilled and well-informed about the PPIUCD procedure itself. Given the comparatively complex nature of the insertion process, they expressed that providers need training or supervision in this practice.

##### Complications

One of the barriers identified by the healthcare providers was potential complications following the insertion of postpartum IUCD. These complications included missing threads, abnormal bleeding, expulsion, and rare complications.

“I have my own experience; I used to have heavy bleeding and abdominal pain while using it. I conceived a baby even while I was using IUCD.”

HCP4, Nurse, Karnali province

##### Preferred alternatives

The healthcare providers identified women's preferences for other contraceptives as a significant barrier to implementing PPIUCD. They reported that women favoured implants as long-acting contraceptives or other hormonal short-acting contraceptives over IUCD use.

##### Beliefs about consequences

Healthcare providers who were practising PPIUCD insertion also expressed concerns about post-insertion consequences, especially when women suffered from complications or could not locate the IUCD thread. In these instances, women had to either return to the health facilities where they received the service or attend an advanced health facility as basic health facilities are not equipped with ultrasound or a skilled workforce to assess the status of the IUCD. When women returned to the facility, the doctors who had inserted the IUCD needed to redirect them to the radiology department or the labour room amid other duties. There were instances when participants experienced, witnessed or heard of rare complications such as uterine perforation or dislodgement of PPIUCD, and accusations of professional negligence.

##### More urgent care takes priority

The healthcare providers reflected that childbirth remained a priority for those assisting in deliveries. They stated that during childbirth, highest priority is given to the management and prevention of birth complications, leaving limited time to counsel women about the PPIUCD.

“I have had instances when I had to leave OPDs (outpatient department) to take patients to the ward, where the ultrasound machine was, but it was not feasible to do every time.”

HCP12, Doctor, Koshi province

#### Theme 3: inadequate capacity and capability

##### Inadequate staff and high workload

Both healthcare provider participants and policy participants reflected on the high workload at maternity services, which reduced the time and effort available for providing counselling and hindered the delivery of postpartum family planning services. They expressed that staff are not adequately allocated based on workload.

“The truth is the staff is low currently. There is a rush in the antenatal, postnatal, labour and gynaecology wards during duty hours. Sometimes, two staff members also have to handle one shift.”

HCP 6, Nurse, Madhesh province

##### Inadequate training

Both healthcare and policy participants frequently cited inadequate training as a barrier to implementing PPIUCD.

“In addition, we lack trained staff to deliver services. It seems to me that we don’t have enough training frequently.”

HCP6, Nurse, Madhesh province

##### Variability in family planning counselling practice

Participants expressed that PPIUCD, being a newer intervention, demanded more time to help women understand it. They highlighted barriers such as high workload, limited time and insufficient trained workforce in the maternity ward and during antenatal care, lack of private space for counselling, designated counsellors and their responsibility to prioritise critical health conditions during the birthing process. Healthcare providers also expressed the need for a context-specific counselling approach rather than adopting a uniform approach for all women.

“We provide counselling especially to each case of caesarean section and multigravida women.”

HCP11, Doctor, Gandaki province

##### Attitudes of healthcare providers

This subtheme emerged from the policy participant interviews. The policymakers reflected that healthcare providers’ practice preferences are affected by the presumption of poor hygiene and their interest towards more complex and advanced procedures rather than family planning or routine maternity care.

#### Theme 4: inadequate investment and priority

##### Limited resourcing and/or preparedness of health facilities

Alongside inadequate staffing, a high workload, inadequate training, and insufficient, minimal, or no financial incentives, participants also expressed concerns about the lack of space for counselling, counsellors, and equipment to deal with potential complications. They reported that many health facilities do not provide this service.

“We also need to create a dedicated space for daily counselling. What we don't have is adequate human resources, time and a system to motivate them.”

HCP9, Nurse, Lumbini province

##### Oversight issues

Participants expressed concern about the inadequate level of supervision and monitoring for PPIUCD. They stated that once staff were trained, a review process was needed to assess whether service delivery had occurred.

##### Cost and funding

Participants highlighted that the cost of PPIUCD was subsidised, and consequently, patients initially received the PPIUCD service at no cost. However, women were required to pay for extra care for follow-up and complication management. These costs were particularly high as they needed to attend the facility where they received the service or other advanced facilities. This led to costs relating to travel, accommodation and other indirect costs such as loss of wages. Policy participants, in particular, revealed how funding is reduced in the family planning sector.

“But the main setback is the loss of copper-T thread, and when they don’t feel or see the IUCD thread, the recipients are required to pay around 3–4 thousand for an ultrasound procedure to find if the copper-T was dislodged and for its removal. … … Initially, it seemed free to patients for insertion, but they perceived it as not free when they faced complications.”

HCP12, Doctor, Koshi province

##### Little appetite for change

Alongside low recipient demand, both participants noted that PPIUCD was not given adequate attention due to a lack of specified targets or goals for meeting key performance indicators in relation to postpartum family planning services.

“We don’t have any target such as PPIUCD prevalence rate to achieve”

HCP1, doctor, Sudurpaschim province

##### Incentive provision

The healthcare providers considered there were no or minimal monetary incentives for delivering PPIUCD.

#### Theme 5: contextual factors

##### Spouse, hygiene and endemic infection considerations

Participants identified contextual factors as barriers to implementing PPIUCD. They noted that many women declined PPIUCD as their husbands mostly worked as migrant workers outside the country, making them absent from regular sexual contact at home. The participants expressed their hesitancy due to poor hygiene conditions. Similarly, their perception that pelvic inflammatory diseases are common among women in Nepal also discouraged them from recommending PPIUCD. The endemic prevalence of HIV/AIDS in certain geographical regions, especially in the western region of Nepal, was another reason for hesitation and, instead, a major recommendation of use of barrier methods of contraception.

### Perceived enablers to implementing integrated PPIUCD services

We identified three key enabler themes, as summarised in Table 4. Illustrative quotes for the enabler themes are provided in Table 5, and a full list of quotes is provided in Supplementary Table S5.

**Table 4:** Identified codes, enabler themes, subthemes, and mapping to theoretical frameworks.

Codes	Themes	Subthemes	TDF	CFIR domains: constructs
1 Pregnancy during the postpartum phase is common 2 Unintended pregnancy	Reducing unmet need for contraception	Suboptimal use of contraceptives	Environmental context and resources (contextual need); Goals; Intentions; Beliefs about consequences; Knowledge	Innovation domain: Relative advantage Individuals domain: Recipients need, motivation, opportunity; Deliverer's motivation, opportunity Inner setting: Relative priority; Mission alignment
3 PPIUCD use avoids unintentional pregnancy		Unintended pregnancy		
4 High abortion rate 5 Women use medical abortion from pharmacies 6 Women coming to hospital for blood transfusion post abortion		High use of abortion for unwanted pregnancy		
7 Less frequent pregnancies result in better maternal health 8 Increased birth spacing 9 Less frequent pregnancy result in better neonatal health 10 Women will get time to accomplish education and occupation	Integrated PPIUCD insertion service is beneficial	Integrating PPIUCD has many benefits (Appropriate reproductive behaviour; Perceived benefit to multiple stakeholders; Promotion of women's autonomy and wellbeing, and neonatal wellbeing)	Beliefs about consequences; Social influences	Innovation: Innovation design (compatible post-childbirth); Relative advantage Individuals domain: Recipients' need and opportunity; Available resources
11 IUCD is non-hormonal 12 No hormone related side effects 13 It is long-acting 14 It is effective and reversible 15 Healthcare providers can assess the reproductive tract during this process		PPIUCD has high relative advantage		
16 The expulsion rate is lower in the immediate insertion of IUCD 17 Opportunity to meet reproductive-aged women 18 Women don't have to show their reproductive part again 19 Control is in recipient's hand and providers' effort		Opportunity during postpartum period		
20 Free supply of contraceptives from government and free basic service 21 Good partnership between NGOs and government 22 Support of external development partners	Policy and partnership	Availability of equipment and resources	Goals; Environmental context and resources; Behavioural regulation	Outer setting: Policies and laws; Partnerships and connections
23 Family planning is priority program of Nepal 24 Safe-motherhood policy and constitution		Alignment with reproductive health policies and guidelines		

Note: CFIR, Consolidated Framework for Implementation Research; IUCD, Intrauterine Contraceptive Device; NGO, Non-governmental organisation; PPIUCD, Postpartum Intrauterine Contraceptive Device; TDF, Theoretical Domains Framework.

**Table 5:** Examples of illustrative quotes for enabler themes.

Enabler themes	Illustrative quotes
Reducing unmet need for contraception	“We should be more focused on the province 2. I am seeing many women with multigravida 5,6,7 on a day-to-day basis, and I feel sad for them. They look lean, thin, skinny; almost all women are anaemic here.” -HCP10, Medical officer, Madhesh province “Today, I met a 49-year-old pregnant lady with a 16-year-old son. She still didn't know that she should use such a device because she never had an unplanned pregnancy before.” -HCP8, Doctor, Bagmati province “Obviously, fertility can return anytime, and when they don't use any family planning devices, they go through unplanned pregnancy” -HCP8, Doctor, Bagmati province
Integrated PPIUCD service is beneficial	“If PPIUCD is used, it will benefit the whole nation, including the patient parties and healthcare providers. Everyone benefits with family planning coverage.” -HCP7, Nurse, Bagmati province “Primiparous women are the number one beneficiary from the integrated family planning services because they can decide about the spacing for next baby and when they can have babies, how long they can breastfeed.“-HCP4, Nurse, Karnali province “PPIUCD is one of the best non-hormonal methods. Implants, Depo-Provera have many side effects, such as bleeding and obesity. PPIUCD is good as it doesn't have side effects. But it is hard to make recipients understand.” -HCP3, Nurse, Sudurpaschim province “Compared to other methods, it's a long-term temporary method, effective, has low failure rate.” -HCP2, Doctor, Karnali province “The IUCD is inserted through the vagina. This also carries advantages as sexual diseases can be identified, cervical polyps, erosions, treatment can be done.” -HCP9, Nurse, Lumbini province “In my opinion, it is very important to integrate maternity care and family planning. If family planning is provided along with maternal healthcare, if we start family planning services during antenatal care of MCH [maternal and child health], we would expect to get good results … … At that period, the counselling can be effective as women may not want to conceive immediately; they may want to have some spacing.” -PP4, Family planning inspector, Bagmati province “When we compare this with other options, it's non-hormonal, while many other options are hormonal. With this, the side effects caused by hormones are prevented. Other hormonal options have short-term effects compared to this one. It is easier if you want to take it out or discontinue it. Because of that, I think it is better.” -PP10, Nursing administrator, Central level
Policy and partnership	“Postpartum family planning, delivery services, cafeteria approach counselling are basic things. This is given by the constitution as a basic right as well (referring to ‘access to family planning service’). It should continue.” -HCP14, Doctor, Lumbini “Our health facilities don't have to buy materials for family planning; it is subsidised 100% by the Nepal government, both central and provincial level of the government provide the family planning commodities.” -PP1, Public Health Officer, Koshi province “The family welfare division itself has also taken initiatives to initiate private hospitals in providing family planning services and have been providing training to the staff.” -PP7, Director, Central level “Now, many other organisations are also working on it, like professional, non-governmental organisations like Marie Stopes, Family Planning Association.” -PP8, Nursing Administrator, Gandaki province

Note: IUCD, Intrauterine Contraceptive Device; PPIUCD, Postpartum Intrauterine Contraceptive Device.

#### Theme 1: reducing unmet need for contraception

Participants perceived the integration of counselling for postpartum family planning and PPIUCD insertion as an important strategy for addressing the identified unmet need for contraception among women of reproductive age.

##### Suboptimal use of contraceptives

Participants perceived there to be suboptimal use of contraceptives among Nepali women, leading to unwanted pregnancies and abortions. They considered that the integration of PPIUCD with maternity care could prevent unwanted pregnancies, abortions and reduce maternal and neonatal mortality and morbidity. They reflected that family planning integration should remain a priority for Nepali women. They suggested a need to revamp existing family planning to focus on and customise the implementation of family planning services.

##### Unintended pregnancy

Participants reiterated the high prevalence of unintended pregnancy, implying a need for adequate family planning provision.

##### High use of abortion for unwanted pregnancy

Participants noted the high prevalence of abortion among Nepali women. Healthcare providers also shared instances when women visited health facilities only after excessive post-abortion bleeding or other complications.

“We have got abortions during the late postpartum phase. They come for abortion with three months pregnancy with five months old baby in their arm.”

HCP4, Nurse, Karnali province

#### Theme 2: integrated postpartum intrauterine contraceptive device insertion service is beneficial

##### Integrating PPIUCD has many benefits

Participants perceived that postpartum family planning benefits women, their families, communities, the health system and the country overall. They reflected on the positive impact on women's autonomy that could be achieved through family planning and improving maternal and neonatal survival and wellbeing.

“I want to give an example of myself. When I had my first baby; I was a young girl. But I didn’t give birth to second baby until eight years later. And during that duration, I studied nursing diploma, intermediate, prepared for public service commission exams and passed it and progressed in my career. When I got information about the family planning, I used it. and with personal experiences too, I know it is important.”

HCP4, Nurse, Karnali province

##### PPIUCD has a high relative advantage

Participants highlighted various benefits of the PPIUCD, including that it is long-acting, free from systemic hormone-related side effects (relevant to copper IUCD) and highly effective in preventing pregnancy. They also focussed on the positive aspects of this contraception, such as easy removal when required and the quick return to usual fertility after removal of the IUCD, unlike Depo-Provera (medroxyprogesterone acetate).

##### Opportunity during the postpartum period

Participants expressed that PPIUCD service integration provides an opportunity to consult with reproductive-aged women who would otherwise not have come to the healthcare centre. Similarly, they also perceived it as a timely opportunity to assess the overall gynaecological and reproductive health of women by assessing any abnormalities such as cervical polyps, and lesions during the process of IUCD insertion. The participants also reflected that women are receptive to counselling after recent pregnancy experiences and could decide upon plans for subsequent pregnancy and their reproductive goals. The policy stream also reflected that the uptake of the PPIUCD is dependent on the amount of effort invested by the healthcare providers and the desire of the recipients.

“The biggest advantage of PPIUCD is that the women of reproductive age come in contact with the hospital during hospital delivery, so this can increase the contraceptive prevalence rate by increasing acceptance and insertion rate increase.”

HCP12, Doctor, Koshi province

#### Theme 3: policy and partnership

Participants expressed that some of the structural and governance requirements are already in place as policy for family planning service provision in Nepal has been prioritised. Participants agreed that inclusion of family planning services as a free basic health service under the Reproductive Health Act and Nepal's constitution is an excellent impetus for service delivery. The healthcare providers stated that health facilities usually had a good supply of family planning devices, though there were occasional out-of-stock issues due to disruptions in the inventory chain. The participants also reflected that key partner organisations are dedicated to the family planning programme in Nepal.

“Postpartum family planning, delivery services, cafeteria approach counselling are basic things. This is given by the constitution as a basic right as well. It should continue.”

HCP14, Doctor, Lumbini province

##### Mapping of themes to CFIR and TDF domains

**Fig. 3:**
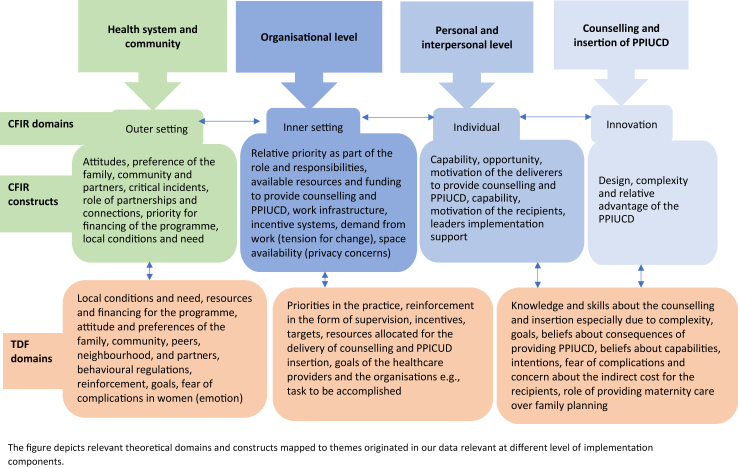
**Summary theoretical domains and constructs mapped to the themes relevant at different level of implementation**. The figure depicts relevant theoretical domains and constructs mapped to themes originated in our data relevant at different level of implementation components.

The emergent themes were mapped to the domains of CFIR and TDF, as shown in Table 2, Table 4 The identified themes were related to twelve TDF domains, including knowledge (knowledge of providers about the intervention), skills (counselling skills, insertion procedure, complication management), beliefs about capabilities, and beliefs about consequences (to women and providers relating to complications, cost) to increase confidence in healthcare providers to deliver integrated PPIUCD. When the identified themes were mapped to CFIR, four domains were most salient, including the outer setting (i.e., the community or the health system overall, partnership among implementing partners), inner setting, individuals domain and the innovation domain. We also found that these two theoretical frameworks helped to interpret the key barrier and enabler factors that should be considered for implementing and scaling up the PPIUCD integration in maternity care (Fig. 3). For instance, the knowledge, skills, and capabilities domains of the TDF are explained by the complexity of the PPIUCD insertion process (innovation domain).

## Discussion

Our study uniquely explored the perspectives of healthcare providers and policymakers to identify the barriers and enablers to successfully integrate counselling for postpartum family planning, and insertion of PPIUCD within maternity services in Nepal. The study identified five main barriers from a health service provision perspective, including care recipients’ limited awareness of and interest in PPIUCD leading to low demand, PPIUCD insertion procedure and contraceptive preferences, inadequate capacity and capability, insufficient investment and priority, and contextual factors. The identified enablers, including recognition of the urgent need for contraception, the benefits of the integrated PPIUCD service, and supportive policy and partnership, can be leveraged to facilitate the integration of these services. The identified factors have significant implications for the overall integration of family planning in maternity care settings, although some themes were specific to PPIUCD uptake. The study also found a subtle difference in the views of healthcare providers and policy participants regarding PPIUCD integration. Healthcare providers emphasised the complications and outcomes of the procedures, while policy participants emphasised the importance of healthcare providers' active involvement in promoting the use of integrated postpartum contraceptive services.

### Findings in context with existing literature

Most previous studies on this topic in Nepal^28^^,^^45^ have assessed barriers to the uptake of postpartum family planning counselling and PPIUCD from the recipients' perspectives. A study by Puri and colleagues which explored providers’ perspectives, identified factors such as inadequate human resources, work overload, and the lack of private space for counselling, logistics and information education and counselling materials.^31^ We believe this is the first study in Nepal to explore the perspectives of healthcare providers and policymakers using in-depth qualitative analysis, to inform potential future system-level implementation and scale-up activities.

To place our findings in a broader context, we looked for studies focussing on healthcare providers' perspectives in similar settings and in developed regions. Similar to our findings, a survey of 344 healthcare providers in India found that women rejected PPIUCD due to factors such as anticipated health problems or side effects, myths, rumours, fears or misconceptions about the product, and opposition from husbands and family members.^46^ Perceived low demand was also identified as a barrier by providers in the USA for IUCDs.^47^ Healthcare providers in Rwanda also highlighted misconceptions as barriers requiring counselling for PPIUCD.^48^

The providers' perspectives we identified align closely with the recipients' perspectives as explored in previous studies in Nepal,^28^^,^^29^ India^49^ and Ethiopia,^50^ which include barriers such as fear of pain, bleeding and other complications, husband's refusal, and lack of family support. This shows importance of other contributing factors such as family, peer, and societal influences affecting PPIUCD use despite postpartum mothers' positive attitudes towards PPIUCD in Nepal.^28^ There is an urgent need for health education programmes targeting community, family and women and the incorporation of standard counselling sessions into maternity care to empower women and family to make informed choices about family planning and contraception.^51^ These programmes are particularly important within the context of a patriarchal society such as that of Nepal,^52^ where there may be additional influences on women's decision making.

This study also explores significant barriers relating to the supply side, including inadequate capacity, given the PPIUCD procedure is complex compared to other contraceptives, and variability in family planning counselling practices. Research in Nepal has shown a gap in healthcare providers' knowledge, attitudes and practices regarding PPIUCD.^53^ Additionally, suboptimal counselling for postpartum family planning has been reported due to various factors, including a crowded environment, limited time with providers, non-availability of providers, long waiting times, and lack of information education and counselling materials related to family planning.^45^ Similarly, studies from the USA^47^^,^^54^ and a narrative review^55^ also highlighted a lack of training and staff unavailability as barriers to providing PPIUCD. In addition, a lack of integration of family planning within maternal and child health service delivery at the health system level has been noted as a significant missed opportunity for improving contraceptive uptake.^56^ This emphasises a need for greater investment in enhancing the capability and capacity of healthcare providers (i.e. their skills, knowledge, beliefs about capacities and capability) through training, continuing medical education and regular oversight as a part of human resource management.

We also identified some concerns regarding perceived poor hygiene and pelvic inflammatory diseases (PID), with potential implications for whether women were considered eligible for PPIUCD insertion. While these concerns are important, poor hygiene is not a risk factor for PID, nor does it affect women's eligibility for PPIUCD insertion.^19^ In addition, contextual barriers such as prevalent HIV infection and migrant working conditions of spouses also warrant the need for reliable contraceptives, making PPIUCD a relevant option along with the use of barrier methods.^19^

It is evident that healthcare providers and policymakers in Nepal are supportive of offering PPIUCD services due to the benefits of PPIUCD integration, such as the prevention of unwanted pregnancies and abortion, and its relative advantages over other contraceptives. However, fear of potential complications^57^ and inadequate infrastructure to address these complications and the costs associated with managing complications prevented healthcare providers from more fully promoting the use of PPIUCD for women. Despite family planning being considered a priority health programme and its provision as a free service in Nepal,^6^ healthcare providers reflected that the accessibility of these services is inadequate. Furthermore, participants emphasised that the current level of investment is insufficient to adequately prepare health facilities to deal with potential complications.

A strength of this study was the use of two theoretical frameworks to robustly guide our inquiry and analysis and minimise missing important factors relevant to implementation. We also included participants who were directly involved in delivering the health services of interest (including representation from medical specialists and nursing and midwifery practitioners with a breadth of clinical experience from newer graduates through to highly experienced professionals) and health-related policymakers, administrators, and managers working in different levels of health facilities and organisations in all provinces of Nepal. Taken together, this is likely to increase the generalisability of our findings for postpartum family planning in Nepal.

Considering the limitations of the study, the interviews were conducted remotely, which might have led to bias by only including participants with access to audio or video conferencing. This could have resulted in the underrepresentation of voices from economically disadvantaged geographical areas, which is important to consider. While this study included a diverse range of healthcare professionals and policymakers from all seven provinces of Nepal and reached theme saturation, we cannot be certain that the participants' views fully represent all perspectives on the factors influencing the implementation of PPIUCD services. We acknowledge that women who would benefit from the integration of PPIUCD with maternal services were not included as participants. Themes pertaining to recipients were derived from healthcare professionals and policymakers and represent their perspectives. We note that previous studies have specifically explored women's perspectives,^28^^,^^45^ and we have compared their findings with our own findings to understand the implications more broadly across the various perspectives. We did not intend to undertake thematic analysis by participant group (as both groups were considered to provide vital and complementary perspectives), although we did identify and report some subthemes that were pertinent to only healthcare providers or policy participants. All interviews were conducted in the native language which provided ease in information sharing, but some meaning may have been lost in translation into English. Additionally, we recognise that the scope of this study was limited to factors influencing postpartum family planning counselling and PPIUCD uptake and we did not consider factors influencing the uptake of other postpartum contraceptive methods.

This study comprises healthcare providers' and policymakers' perspectives, fostering a deeper understanding of relevant challenges to implementation and scale-up of the postpartum family planning services, particularly counselling for postpartum family planning and PPIUCD integration. The identified issues highlight the urgent need for increased investment in Nepal's maternal health facilities to enhance their preparedness and strengthen the capabilities of healthcare providers and health service capacities. Our findings also underscore the need for better community-based information, education and communication activities and targeted health education for pregnant and postpartum women and families. Given prevalent misconceptions about PPIUCD, generating accurate data on post-insertion complications will assist in clarifying women's understanding of this intervention and aid the promotion of PPIUCD integration within maternity care. Furthermore, these research findings also facilitate the integration of other postpartum contraceptive options when deemed appropriate, ultimately enhancing access and informed choices available to postpartum mothers.

## Contributors

PR (1), DAOC, INA, and RB conceptualised the study, and PR (1) managed project administration. All authors assisted in the project administration. PR (1) collected and managed data. PR (1) transcribed the interview verbatim, and PR (1) and SPR translated the transcripts into English. PR (1) and PR (2) coded the data for thematic analysis. RB, DAOC, and INA reviewed quotes and supervised and validated the themes and codes. The manuscript was drafted by PR (1). All authors contributed to the manuscript review and editing. RB, DAOC, and INA supervised the overall research. All authors agreed to submit for publication.

## Data sharing statement

All relevant data are included in the paper and Supplementary Files. The study's audio files and transcripts contain sensitive and personal information about the participants, and cannot be shared under current approvals to maintain confidentiality.

## Declaration of interests

RB receives numerous grants from Australian National Health and Medical Research Council (NHMRC), Medical Research Futures Fund (MRFF), Australian government, HCF Foundation, and Arthritis Australia (provided to institution). RB received UpToDate royalties for chapter on Plantar Fasciitis. RB has also received travel support to attend National Health and Medical Research Council (NHMRC) committee meetings and to give invited talks related to their own research at conferences (unrelated to current study). PR (1) is supported by the Monash International Postgraduate Research Scholarship and the Monash Graduate Scholarship; DAOC is supported by Australian NHMRC Investigator Fellowship (APP2025661). The authors declare no other conflicts of interest.

## Appendix A Supplementary data

Abstract in Nepali
