# Supplementary material for: Clinician and policymaker perspectives on the barriers and enablers to implementing and scaling up integrated postpartum intrauterine contraceptive services within maternity care in Nepal: a qualitative study
Source: Lancet Reg Health Southeast Asia. 2025 May 14;37:100599. doi: 10.1016/j.lansea.2025.100599 (PMC12141544; doi:10.1016/j.lansea.2025.100599)
Supplement: Abstract in Nepali [file mmc1.docx]

Editor Note: This translation in Nepali was submitted by the authors and we reproduce it as supplied. It has not been peer reviewed. Our editorial processes have only been applied to the original abstract in English, which should serve as reference for this manuscript.

**सार/संक्षेप (Summary)**

**पृष्ठभूमि (Background)**

मातृस्वास्थ्य सेवासंगै एकीकृत प्रसवोत्तर परिवार नियोजन सेवाहरू, विशेष गरी सुत्केरी पछाडी प्रयोग गर्न सकिने परिवार नियोजन सम्बन्धि परामर्श र पोस्टपर्टम इन्ट्रायूटेरिन गर्भनिरोधक उपकरण (कपर-टी: नेपाली जनमानसमा प्रसिद्ध नाम वा PPIUCD) प्रयोगले अनिच्छित गर्भधारण र अनिच्छित गर्भधारणले निम्त्याउने जटिलताहरूलाई कम गर्न मद्दत गर्न सक्छ। यस अध्ययनले स्वास्थ्य सेवा प्रदायक र नीति निर्माताहरूको दृष्टिकोणबाट नेपालको मातृस्वास्थ्य सेवा क्षेत्रभित्र यी सेवाहरूको कार्यान्वयन, एकीकरण, तथा विस्तारलाई असर गर्ने कारकहरूको खोजी गरेको छ।

**विधिहरू (Methods)**

यस गुणात्मक (qualitative) अध्ययनको लागि, हामीले विषयवस्तु सम्बन्धि परिपूर्णता (thematic saturation) प्राप्त नभएसम्म नेपाली मातृस्वास्थ्य सेवा क्षेत्रका सातवटै प्रदेशका स्वास्थ्य सेवा प्रदायकहरू र नीति निर्माताहरूसँग टेलिफोन/अनलाइन दुरसंचारको माध्यमबाट गहन, अर्ध-संरचित अन्तर्वार्ताहरू सञ्चालन गर्यौं। सम्भावित योग्य सहभागीहरूलाई सार्वजनिकरूपमा उपलब्ध इमेल ठेगानाहरू, व्यक्तिगत सम्पर्कहरू, स्नोबल स्याम्प्लिंग (snowball sampling), र सामाजिक मिडिया विज्ञापनहरू मार्फत आमन्त्रित गरिएको थियो। उत्तरदाताहरूलाई योग्यताको लागि मूल्याङ्कन गरियो र योग्यता सन्तुष्ट गर्ने उत्तरदाताहरूलाइ अनुसन्धानमा समावेश गरियो। दुईवटा सैद्धान्तिक ढाँचाहरु, कन्सोलीडेटेड फ्रेमवर्क फर इम्प्लिमेंटेशन रिसर्च (Consolidated Framework for Implementation Research) र थियोरेटीकल डोमेनस् फ्रेमवर्क (Theoretical Domains Framework) फ्रेमवर्कले हाम्रो अनुसन्धान र विश्लेषणलाई मार्गदर्शन गर्यो। हामीले मौखिक अन्तर्वार्ताहरुलाई शब्दश: लिपिबद्ध गर्यौ, र उक्त प्रतिलिपिहरूलाई अंग्रेजीमा अनुवाद गर्‍यौं र विषयगत विश्लेषण (thematic analysis) को मद्धतले विश्लेषण गर्‍यौं।

**निष्कर्षहरु (Findings)**

छब्बीस अन्तर्वार्ताका आधारमा, हामीले पाँच प्रमुख अवरोध विषयहरू पहिचान गरेका छौं, जुन सेवाग्राहीहरु (महिला, परिवार तथा समाज), स्वास्थ्य सेवा प्रदायकहरू, स्वास्थ्य संस्था र स्वास्थ्य प्रणालीसँग सम्बन्धित छन्।यसमा तलका पाँच विषयहरू समावेश थिए: (१) सेवाग्राहीहरूमा PPIUCD सेवाको अपर्याप्त चेतना र उक्त सेवाप्रति कम इच्छा, (२) PPIUCD-सम्बन्धीत विशेष मुद्दाहरू, (३) PPIUCD सेवा प्रदान गर्न अपर्याप्त क्षमता र सामर्थ्य, (४) अपर्याप्त लगानी र प्राथमिकता, र (५) सान्दर्भिक कारकहरू जस्तै सेवा प्रदायकहरुको पेभ्लिक इन्फ्लामेटरी डिजिज र सरसफाई सम्बन्धि विचार। स्वास्थ्य सेवा प्रदायकहरू र नीति निर्माताहरु बीच केही फरक दृष्टिकोणहरु पनि पाइयो। नीति निर्माताहरुलाई प्रतिनिधित्व गर्ने सहभागीहरूले स्वास्थ्य सेवा प्रदायकद्वारा परामर्श र PPIUCD उपलब्ध गराउने प्रयासको कमीलाई जोड दिए, जबकि स्वास्थ्य सेवा प्रदायकहरूले PPIUCD- सँग सम्बन्धित समस्याहरू (जस्तै, कपर-टी राख्ने प्रक्रियाको जटिलता) लाई अवरोधको मुख्य कारणको रूपमा पहिचान गरे। स्वास्थ्य सेवा प्रदायक र नीति निर्माताहरू दुवैले PPIUCD लगायत प्रसवोत्तर (postpartum) गर्भनिरोधक सेवाहरूलाई एकीकृत गर्नमा अपर्याप्त लगानी र प्राथमिकतालाई अर्को महत्त्वपूर्ण कारकको रूपमा पहिचान गरे। सहभागीहरूले प्रभावकारी एकीकृत परामर्श र गर्भनिरोधक सेवाहरू लागू कार्यन्वयन गर्न तत्काल आवश्यकता रहेको संकेत गरे।

**व्याख्या (Interpretation)**

नेपालमा मातृसेवामा एकीकृत प्रसवोत्तर परिवार नियोजन सेवाहरू, विशेष गरी पोस्टपर्टम इन्ट्रायूटेरिन गर्भनिरोधक सेवाहरु कार्यन्वयन गर्न र विस्तार गर्ने बहु-स्तरीय अवरोधहरूलाई सम्बोधन गर्न ठूलो लगानी आवश्यक छ। सेवाग्राही र समुदायका लागि स्वास्थ्य शिक्षा, सेवा प्रदायकहरुको क्षमता निर्माण (हेरचाह प्रदायकहरू सक्षम छन् भन्ने सुनिश्चित गर्दै), र स्वास्थ्य संस्थाहरूको स्तरीकरणमा प्राथमिकता दिनुपर्छ।
