# Supplementary material for: Clinician and policymaker perspectives on the barriers and enablers to implementing and scaling up integrated postpartum intrauterine contraceptive services within maternity care in Nepal: a qualitative study
Source: Lancet Reg Health Southeast Asia. 2025 May 14;37:100599. doi: 10.1016/j.lansea.2025.100599 (PMC12141544; doi:10.1016/j.lansea.2025.100599)
Supplement: Supplementary Figure [file mmc2.docx]

**SUPPLEMENTARY INFORMATION**

**Supplementary Figure S1. Data analysis process.**

Step 1: familiarisation with data

Step 2: generating initial codes

Step 3: identification of subthemes and themes from codes

PR(1) was responsible for generating initial codes. PR(2) also generated codes (deidentified data) independently (for reliability and checking subjectivity). The codes were verified by INA, DAOC, RB thoroughly reviewing the quotes.

PR(1) grouped codes to generate themes based on similarities of the codes and DAOC, INA and RB verified the themes.

PR(1), INA, DAOC, RB identified subthemes when applicable.

Theme and subtheme names were finalised by PR(1), DAOC, INA and RB. Exemplar quotes were selected to reflect codes and subthemes by PR, DAOC, INA and RB.

PR(1) transcribed interview verbatim. PR(1) and SPR translated transcripts into English.

Step 4: mapping of themes and subthemes to the constructs and domains of theoretical frameworks

PR(1), DAOC, INA and RB mapped themes and subthemes to relevant constructs and domains of the Consolidated Framework for Implementation Research and Theoretical Domains Framework.

Note: DAOC – Denise A. O’Connor; INA – Ilana N. Ackerman; PR(1) – Pramila Rai; PR(2) – Pabitra Rai; RB – Rachelle Buchbinder; SPR – Surya Prasad Rimal
