# Supplementary material for: Clinician and policymaker perspectives on the barriers and enablers to implementing and scaling up integrated postpartum intrauterine contraceptive services within maternity care in Nepal: a qualitative study
Source: Lancet Reg Health Southeast Asia. 2025 May 14;37:100599. doi: 10.1016/j.lansea.2025.100599 (PMC12141544; doi:10.1016/j.lansea.2025.100599)
Supplement: Supplementary Information [file mmc3.docx]

| **SUPPLEMENTARY INFORMATION**  **Interview guideline for healthcare providers** |
| --- |

**Opener:** Thank you so much for making the time to share your insights with me. The purpose of this interview is to understand the factors influencing implementation and scale-up of the integration of postpartum family planning services within maternity services in Nepal. When thinking about postpartum family planning services, we are referring to providing family planning counselling during the antenatal, intrapartum and postnatal periods and the insertion of an intrauterine contraceptive device (IUCD) immediately after birth of the baby (PPIUCD: postpartum intrauterine contraceptive device) in an integrated manner through usual maternity care. There are no correct answers, and your thoughts and opinions will help us to understand the context and what would be required to effectively implement and scale-up this intervention in hospitals or healthcare facilities in Nepal.

The interview will last around 45-60 minutes.

- Please let me know at any point, if you would like to take a break, if you prefer not to answer a question, or stop the interview,

- The interview is being recorded, but your identity will be strictly confidential and you will have a unique identifying number

- Do you have any questions before we start?

1. Can you tell me about the family planning services that are currently provided in your healthcare organisation?
2. What family planning services are provided to women postpartum?
3. Can you tell me about your role in providing contraceptive counselling and delivery of PPIUCD to women? During antenatal care, after birth of baby, postnatal period?

| Domains | Questions and prompts |
| --- | --- |
| 1. Intervention or   Innovation characteristics | - How complicated or easy is the family Planning counselling procedure at the time of maternity care? How effective, costly, safe, reliable, helpful is family planning counselling in your opinion? - In your opinion, how complicated is the PPIUCD insertion? How effective, costly, safe, reliable, helpful are PPIUCD in your opinion? - How does PPIUCD compare to other family planning measures? Why PPIUCD specifically? Are there another family planning measures that women would rather choose after birth of the baby? What kind of changes or alterations do you think you will need to make to the PPIUCD implementation so it will work effectively in your setting? - What is your perception of the quality of the supporting materials, packaging, and bundling of the intervention for implementation? |
| 1. Individual characteristics | **Family planning counselling during maternity care (if their role includes only counselling):**   - Is family planning counselling something that you routinely do as part of your job when women come to visit you for antenatal care, delivery, postnatal care? - How important is it to you to do this intervention? To what extent is doing family planning counselling part of your role as a nurse or SBAs or doctor or obstetrician? - How confident are you that you will practice family planning counselling during maternity care? How confident do you think your colleagues feel about this intervention?   **PPIUCD insertion after birth of the baby (If their role includes only PPIUCD insertion):**   - Is PPIUCD insertion something that you routinely do as part of your job when women come to visit you for delivery, and postnatal care? - How important is it to you to do this intervention? To what extent is doing family planning counselling part of your role as a nurse or SBAs or doctor or obstetrician? - How confident are you that you will provide PPIUCD after birth of a baby? How confident do you think your colleagues feel about this intervention?   **Both:**   - Have you got trainings and updates required to deliver family planning counselling or PPIUCD insertion? - How well does family planning counselling and PPIUCD insertion fit with your existing work processes and practices in your setting? Can you describe how the intervention will be integrated into current processes? Where does this fit in your priority list?   If the provider doesn’t have the role of providing PPIUCD or counselling,   - Why are you not providing these services? What conditions would make you capable of providing these services? |
| 1. Inner settings | What factors might make it challenging for you to provide postpartum family planning counselling or PPIUCD immediately after the birth of a baby?   - How will infrastructure of your organisation affect implementation of the intervention? - How essential are these interventions to meet the need of the women coming to get services from this organisation? - In your opinion, what kind of changes do you think you will need to make to the family planning counselling or PPIUCD insertion so it will work effectively during maternity care? - Are the necessary physical and workforce resources available at your hospital or healthcare facility to provide family planning counselling or PPIUCD insertion along with maternity care? Can you tell me more about that? What would be needed to facilitate family planning counselling at the time of antenatal and postpartum care and to facilitate PPIUCD insertion postpartum? - What kind of incentives are there to help ensure smooth implementation of the family planning counselling or PPIUCD insertion? |
| 1. Outer settings | - Have you heard of any organisation that is implementing postpartum intrauterine devices immediately during postpartum phase in an institutional and integrated manner? - How important is the family planning counselling to pregnant women? What factors influence women's decision to choose family planning? - What barriers will these women and family face to participating in the family planning counselling? - Who might benefit from family planning counselling or delivery of postpartum intrauterine devices? How do you think the women and family will respond to the integrated services? - What factors influence women’s decision to choose PPIUCD? What barriers will these women face to use PPIUCD? who might benefit the most from postpartum intrauterine devices insertion? |
| 1. Process | Does your unit or organisation have any mechanism for obtaining feedback or measuring the progress and quality of the services or implementation? |

1. Do you have any suggestions for your supervisors, organisation to enhance routine delivery of PPIUCD counselling and insertion?
2. What are the important factors to consider when scaling up family planning counselling beyond the current practice or settings? PPIUCD insertion beyond current settings or practice? Do you expect to have sufficient resource or support and actions from supervisors to implement and administer the intervention?
3. Is there anything else you would like to tell me about family planning counselling and/or PPIUCD insertion that you think would be helpful for me to know?’

| **Interview guideline for policymakers** |
| --- |

1. Can you describe the current provision of family planning counselling for women during maternity care? To what extent are health institutions regularly providing counselling and PPIUCD insertion to the pregnant and postpartum women?
2. What roles have you carried or have you been carrying or may be carrying in relation to family planning programmes or services?

| Domains |  |
| --- | --- |
| 1. Intervention or   Innovation characteristics | - How complicated or easy to integrate the family Planning counselling and PPIUCD insertion with maternity care? How effective, costly, safe, reliable, helpful is family planning counselling in your opinion? How effective, costly, safe, reliable, helpful are PPIUCD insertion in your opinion? - How does PPIUCD compare to other family planning measures? Why PPIUCD specifically? Are there another family planning measures that women would rather choose after birth of the baby? - What kind of changes or alterations do you think you will need to make to the family planning counselling and PPIUCD implementation so it will work effectively in your setting? - What is your perception of the quality of the supporting materials, packaging, and bundling of the family planning counselling and PPIUCD insertion for implementation during maternity care? |
| 1. Individual characteristics | - How competent are health providers to provide family planning counselling or PPIUCD insertion along with maternity care? - Are there enough guidance or support or opportunities to update the knowledge and skills through in-service training, workshops, CMEs, supervision? |
| 1. Inner settings | - In your opinion, what factors (personnel, resources, organisational, patient-related) affect the regular provision of PPIUCD counselling and insertion immediately after the birth of a baby? - Do you feel necessity of changes in policy, guidelines, rules, organisational behaviour for successful integration of family planning counselling and PPIUCD insertion in health institutions? What kind of changes? - How prepared are health organisations or your organisation to continue or scale-up the PPIUCD routinely? Resources or skills, trainings, guidelines? - Are your employees or healthcare providers equipped to provide postpartum family planning counselling and PPIUCD insertion? Are there any support system in the current health system that would improve delivery of family planning counselling and PPIUCD insertion? - Have you experienced or are you aware of any problems in supply, procurement, storage of family planning commodities especially IUCD commodities? - How should healthcare providers prioritise this intervention? Is this a priority responsibility of healthcare provider? |
| 1. Outer settings | - Have you heard of any organisation that is implementing postpartum intrauterine devices immediately during postpartum phase in an institutional and integrated manner? - How do you think the women and family will respond to the integrated services? - What factors influence women's decision to participate in the family planning counselling and choice of contraceptives? - How important is the family planning counselling to pregnant women? What barriers will these women and family face to participating in the intervention? - What barriers will these women face to use PPIUCD after the birth of baby? who might benefit the most from family planning counselling or postpartum intrauterine devices insertion? - Who are the key stakeholders (champions, change agent, opinion leaders, formally appointed internal leaders) for planning, implementation of this intervention? Does their involvement influence the implementation of the intervention? |
| 1. Process | - Do you have any plans to integrate postpartum intrauterine device insertion during maternity care in your institution or institutions in Nepal? - Have you planned any framework or plan or taskforce to implement this program? - Do your unit or organisation or health offices have any mechanism for obtaining feedback or measuring the progress and quality of the services or implementation (when asked to health executives)? - What organisations or institutions may be helping in its implementation or scale-up? What communication or information strategy are available for implementation and scale-up of the intervention? |

1. What factors are important for scaling up this intervention beyond current practice and settings?
2. What should be the role of health system or organisations or policymakers for the successful scale-up of this approach?
3. Is there anything else you would like to say about integration of family planning counselling as part of maternity care and PPIUCD insertion?

**Note: CME: Continuing Medical Education; PPIUCD: Postpartum Intrauterine Contraceptive Device; SBA – skilled birth attendant**
