# Supplementary material for: Clinician and policymaker perspectives on the barriers and enablers to implementing and scaling up integrated postpartum intrauterine contraceptive services within maternity care in Nepal: a qualitative study
Source: Lancet Reg Health Southeast Asia. 2025 May 14;37:100599. doi: 10.1016/j.lansea.2025.100599 (PMC12141544; doi:10.1016/j.lansea.2025.100599)
Supplement: Supplementary Tables [file mmc4.docx]

**SUPPLEMENTARY INFORMATION**

**Supplementary Table 1 Consolidated Framework of Implementation Research (CFIR) domains and their interpretation in this study.**

| **Domains** | **Interpretation of the domains in this context** |
| --- | --- |
| Innovation | In our study context, the intervention characteristics include features relating to the postpartum family planning counselling, consent taking for intrauterine devices and postpartum intrauterine contraceptive device (PPIUCD) insertion after childbirth. |
| Inner setting | The maternity or obstetrics departments or units at the hospital or health facilities offering maternity care to pregnant women. |
| Outer setting | Patient needs and resources, policy-making agencies including the ministry of health and population, policy influencing agencies such as Nepal medical council, Nepal nursing council, Nepal medical association, civil society, and nongovernmental organisations |
| Individuals | Any providers responsible for the delivery of maternity care. The individuals vary depending on the types of health facilities. For example, a mix of obstetricians and nurses provides antenatal care in the tertiary level health facilities in Nepal, whereas nurses are the sole providers for antenatal and maternity care in a community level health facility. The individuals also include the policymakers, policy influencers and administrators responsible to regulate activities at health facilities. Women and their partners are the recipients of the intervention. |
| The implementation  process | The activities and strategies used to implement the family planning counselling and PPIUCD insertion. It addresses gaps in the current systems and recommendations for formal implementation or scale-up processes in the future by assessing the needs of the recipients and deliverers, improving context, planning, tailoring strategies, engaging, doing, reflecting on outcomes, and evaluating and adapting the processes. |

Note: PPIUCD – Postpartum Intrauterine Contraceptive Device

**Supplementary Table 2 Theoretical Domains Framework (TDF) domains and their interpretation in this study.**

| **Domains** | **Interpretation of domains in this research context** |
| --- | --- |
| Knowledge | Awareness of the existence of the guidelines and standards relating to postpartum family planning counselling and PPIUCD |
| Skills | An ability or proficiency acquired through practice for providing the postpartum family planning counselling and PPIUCD |
| Social or professional role and identity | A coherent set of behaviours and displayed personal qualities of an individual in a social or work setting to identify one’s and other’s role in implementation of such services |
| Beliefs about capabilities | Healthcare provider’s belief and realisation about one’s capabilities and healthcare environment to provide postpartum family planning and PPIUCD insertion |
| Optimism | The confidence that things will happen for the best or that desired goals will be attained |
| Beliefs about consequences | Healthcare provider’s realisation and belief about the consequences of when postpartum family planning counselling and PPIUCD insertion provided as routine maternity care |
| Reinforcement | Factors reinforcing the probability of adherence to the service provision of postpartum family planning counselling and PPIUCD insertion |
| Intentions | A conscious decision of a healthcare provider to provide postpartum family planning counselling and PPIUCD insertion |
| Goals | Motivating factors and personal goals for healthcare providers to implementing postpartum family planning counselling and PPIUCD insertion |
| Memory, attention and decision process | The ability of healthcare providers to retain information on postpartum family planning counselling and PPIUCD insertion and choose to integrate this service in their practice |
| Environmental context | Any personal or organisation or other environmental circumstances encouraging or discouraging healthcare provider’s development of skills and abilities, competence and behaviour required for provision of this service in their practice |
| Social influences | Social influences such as opinions and practice of colleagues on the service provision |
| Emotion | Emotional feeling towards integration of this service into daily practice |
| Behavioural regulations | Any protocols, referral pathways or regulatory mechanism aimed at integrating postpartum family planning counselling and PPIUCD insertion in routine practice |

Note: PPIUCD – Postpartum Intrauterine Contraceptive Device

**Supplementary Table 3 Action, Actor, Context, Target, Time (AACTT) framework for postpartum family planning service including counselling and PPIUCD.**

| Actor (who) | Context (where) | Action (what) | Target (whom) | Time (when) |
| --- | --- | --- | --- | --- |
| Auxiliary nurse midwives (ANMs) | Health Posts, PHCCs, Birthing centres | Provide family planning counselling in antenatal care, and  Take consent for family planning choice esp. PPIUCD insertion | Pregnant women and family | As implementation begins,  At the time of antenatal visit or with contact with the health professionals  During labour if not in active phase of labour  Postpartum after birth of a baby  PPIUCD insertion: Immediately after the birth of a baby until 48 hours and thereafter 28 days postpartum onwards |
| Staff nurses | Health Posts (HPs), Primary Health Care Centres (PHCCs), Hospitals, Birthing centres (BCs) |  |  |  |
| General doctors/  obstetricians | PHCCs, hospitals, and limited number of health posts |  |  |  |
| Hospital executives/Head of the departments/  Health facilities | All Health facilities  Health facilities, healthcare offices, | Planning, Assuring adequate staff, trainings  Developing protocols  Set standards  Ensure appropriate facilities, regulation, | Health workforce  Health settings | At every phase from planning to implementation |
| Public or health administrators or executives from Non-governmental Organisations | Public Health Offices, NGOs | advocacy, monitoring, evaluation, funding |  | At every phase from planning to implementation |
| Policymakers or  Health system | local, state and central level of government especially Ministry of health and population | Planning, advocacy, funding, monitoring | Health Care facilities, hospitals, Healthcare providers, Policy | Planning, implementation, evaluation, continuation phases |

Note: PPIUCD – Postpartum Intrauterine Contraceptive Device; NGO: Non-governmental organisation

**Supplementary Table 4 Illustrative quotes for the barrier themes identified.**

| **Perceived barrier subthemes** | **Illustrative quotes** |
| --- | --- |
| **Barrier theme 1: Recipients’ limited awareness of and desire for PPIUCD as perceived by health professionals** | |
| Limited awareness | “Awareness is of utmost importance. Females marry at early age and many of them haven’t been to school. I think the main important thing is education; if mothers were educated, they would accept the contraceptive services. For example, they are not even aware of family planning. They often don’t know what their choices are. I met women who have been using pills for more than 10 years. This may be due to a lack of awareness and a scarcity of service providers! But, using long-term hormonal methods can have many side-effects. It may even increase the risk of cancer.” -HCP2  “Family planning counselling is very important, in my opinion. In Nepal, many people don't know about contraceptives; it's very sad to see women getting pregnant despite having a 3-4-month-old baby. I think they had to experience this as they lack enough knowledge or information.” -HCP6  “We need to advertise all available postpartum family planning services, especially PPIUCD. Just as there is routine about when to give Iron and Calcium tablets during pregnancy, family planning component should also be added to the regular antenatal care.” -HCP7  “When everyone in the village is aware and self-motivated, they will come and seek the service; otherwise, they question why they should be doing this when they haven’t done it their whole life. Getting rid of myth is only possible if our low-level (means grassroot level) staff actively educates the local women and their family, the tradition that has been going on since the time of our older generation cannot simply be removed by increasing access” -HCP8  “Today, I met a 49-year-old pregnant lady with a 16-year-old son. She still didn't know that she should use such a device because she never had an unplanned pregnancy before.” -HCP8  “This is an underdeveloped part of Nepal. People are not aware of the birth spacing. They have less knowledge of birth spacing. People are ill-informed and have many misconceptions. They have fear of side effects of all kinds of contraceptives.” -HCP10  “We should also increase public awareness campaigns regarding misbelief and misperceptions, and maybe we can remove them! For instance, a few contraceptives such as condoms referred to by Nepali name, ‘Dhal’, Depo-Provera as ‘Sangini sui’are popular, but nobody talks about Copper T, OCPs [oral contraceptive pills], and implants. This should also be changed a bit. If such things become common talk on media outlets such as TV and radio, other media, patients will not be reluctant when we counsel patients. At the same time, demonstration is also important. We can provide mass education by demonstrating on the mother dummy to make everything clear. Similarly, we can motivate patients during intrapartum and postpartum as well.” -HCP10  “Similarly, awareness and education programs with the use of pictures through mass media will help. People are more into social media and television. We can utilise these platforms. Everybody is on Facebook and most people are the google doctor so we can use this platform as a tool.” -HCP10  “The main thing is that the public should be aware of the service, and we need to increase the public awareness because family planning is provided through health services, and that organisation rarely advertises. People don’t know the existence of PPIUCD service” -HCP11  “Another thing is awareness about contraceptives. Women have heard of the copper-t, but very few women are aware of this procedure, PPIUCD. They all know Depo-Provera (uses the common name Sangini sui), tri-monthly injection, pills, condom, and even copper-t. However, they don't understand that postpartum IUCD is the insertion of copper-t during the postpartum phase. That’s why counselling is very important. Their acceptance depends on how much time and information the counsellor can give.” -HCP12  I feel that people are not aware that it is safe or that they are unaware it is beneficial to use. We haven’t been able to penetrate the community with a positive message regarding this service.” -PP1  “Our target is the middle class, low-income class, who face many limitations, knowledge being one. Another thing is that if they have a problem of knowledge, they also have a problem of attitude.” -PP3  “Some women become pregnant without the return of menstruation. They may not know they may get pregnant even without the return of menstruation. I see that we must create demand by finding out the reasons and bringing awareness to the community level.” -PP4  “Maybe we need an awareness campaign to raise awareness about how a deterioration in one woman’s health today can subsequently affect whole family’s health tomorrow. To internalise that their problem is a common problem of the family, we raise such issues to sensitise participants during the training and seminar for women.” -PP5    “We have also perceived a knowledge gap. We have encountered women who had conceived a child when the youngest was still under a year old. They have the conception that they don’t need to use any contraceptives if their menstruation is not returned.” -PP7  “If you look at the functional knowledge of family planning among clients, for example, a significant number of people are unaware of how long-acting contraceptives work, and what it does. DHS [demographic and health survey] shows that 99% of those who answered that they have heard about family planning, but if you look at the functional knowledge, it is not that high. The condition hasn’t changed much in the last 10 years.” -PP7  “We are providing service by keeping one dedicated midwife. Counselling is important, even prenatal counselling is important. With the provision of midwifery-led antenatal care, the level of counselling may improve. The importance of counselling should be understood by all doctors and nurses, and everyone should give it.” -PP8  “The first thing they need to know is there is something they can use immediately after delivery. They should know about it…… We need to engage the providers and create demand together. We need to increase the awareness program. Community awareness should be increased now” -PP9  “Similarly, there is lack of awareness, but I think first and foremost is that women seem to shy away from showing their private parts, so I think it's underused.” -PP10  “Now people do not know that they can get pregnant even in perimenopausal age. If they reach 45 years old and become old, they say it won't happen now, but I can get pregnant even at that time and we are investing in that too.” -PP11  “Now we are giving free service, we are sending mobile teams to some places, but them not coming to take this service even when it is free means that one is not fully conscious about it, not aware of what will happen after using it, of what to do if something happens.” -PP11  “We have not been able to create awareness among our clients that family planning can be used even in the postpartum phase of that level, so it is very low.” -PP12 |
| Misconceptions | “There are rumours about IUCD getting lost once placed in the uterus. They have a misconception of IUCD causing cancer……They have concerns that IUCD as some foreign body can cause difficulties……. Women think they are weak during the time of postpartum, and putting PPIUCD delays their recovery.” -HCP3  “The mannequin models could have assisted in the counselling process. We could demonstrate to women where IUCD is kept; we could have busted the myths about IUCD showing why it wouldn’t directly reach the heart.” -HCP4  “They believe that IUCD will fall out of the uterus, and it will be uncomfortable for the husband.” -HCP6  “They complain that it is uncomfortable for the husband during a sexual relationship. There are many myths in Nepal.” -HCP7  “They believe that family planning is only necessary when they resume menstruation. That is why patient compliance is very low. Nowadays, the acceptance towards contraceptives has increased due to the efforts of healthcare providers.” -HCP8  “Women also believe the rumours that it creates wounds in the uterus and causes cancer. They also mentioned that sometimes it may perforate and go to other organs, unusual bleeding, irregular menstruation.”-HCP9  “But, mainly the patient themselves declines mostly during counselling as they think it is a nuisance as they need to get rest postpartum.” -HCP9  “I also found one rumour recently as I was taking a history of contraceptives, some women reported that they were not using any contraceptives because they believed that the medicine, they are having for hypothyroidism can prevent conception.” -HCP9  “After counselling, some patients wanted to use it, but some raised concerns of what they heard about complications such as loss of thread, displacement to the intestine.” -HCP10  “Clients believe that IUCD can go beyond uterus piercing uterus wall. Even though we say that’s not the case always, they mostly prefer implants…. But there were some myths and misperceptions such as IUCD should be kept for 10 years and it can be taken out only after 10-12 years, it cannot be taken out immediately.” -HCP13  “There is a rumour of IUCD dislocating to internal organs. So, we also suggest using implants, Depo-Provera.” -HCP13  “There are many misperceptions about IUCDs as well, we have heard people saying that IUCD can move and reach other internal organs, pierce the uterus” -PP2  “Mainly, it may be due to some kind of myth or some kind of understanding that it is something we keep in women’s body, and they might have apprehension about it damaging their uterus.” -PP5  “And they must have heard about some of the complications where the IUCD had to be removed performing laparotomy. It must have given a bad message about the IUCD.” -PP5  “The myths such if that IUCD penetrates the heart, causes cancer, Depo-Provera makes women fatter also prevent them from using contraceptives.” -PP6  “They have conception that they don’t need to use any contraceptives if their menstruation is not returned. They are not well counselled that if they are not doing exclusive breastfeeding, there is a high chance of getting pregnant again even when the menstruation is not returned.” -PP7  “There are many misconceptions. Some say it comes out or doesn’t stay in place. Some believe it pierces the uterus and, reaches the heart and is fatal. There is also a misconception that it makes a wound in the uterus, but women will accept it if counselling is done for that from antenatal.” -PP8  “Misperceptions must be busted as there are misconceptions among women and general people spread by word of mouth that IUCD reaches the heart or other organs.” -PP9  “Women also believe that they are protected from unsafe sex until six months of breastfeeding. Along with that, there are other things as well such as myths, myths if placed in the uterus, that thread also affects the sexual experience, cause wounds, comes out etc.” -PP10  “There is still a belief that some people who are breastfeeding in the postpartum phase will not get pregnant for two years. Now, people do not know that they can get pregnant even in perimenopausal age. They say it won't happen now because they are already 45 years old and old, but they can still get pregnant even at that time, and we are investing in that too.” -PP11  “There are many myths about it.” -PP12 |
| Attitudes | “They don’t prefer IUCD because Women don’t want to show their personal body part due to shyness. Even I feel shy as a female.” -HCP3  “But for people like us, who have high workload, who should be in the field, using PPIUCD is uncomfortable.” -HCP4  “Even we hesitate to show our private parts even if it is for a medical procedure” -HCP4  “It is not preferred even by educated people. Even the PPIUCD users who have used previously came to remove it and shift to other kind of contraceptives.” -HCP4  “It is not difficult to get written consent once the patients agree to receive the service.” -HCP6  “When we ask women the reason for not using contraceptives within 1 year. They explain their decision based on their experience as they didn’t conceive child for 2 years after birth of previous child.” -HCP9  “After counselling, some patients wanted to use it, but some used to raise concerns of what they heard about complications such as losing of thread, displacement to the intestine.” -HCP10  “When young unmarried girls talk about family planning, they are scorned for talking about it.” -PP9  “I had provided PPIUCD to only three women when I took training in Bheri zonal hospital, and after I joined here, no women prefer it.” -HCP4  “IUCD is not preferred, and they don’t think it is a good choice.” -HCP7  “They come to OPD and have 5 minutes of consultation, which won’t change anything in their attitude.” -HCP8  “But the most important thing is a matter of convenience. Many women are using implants because they are placed in the arms; they don't have to expose their private parts. It’s easy for both women and care providers. Providing and using Depo-Provera and pills are also convenient.” -HCP9  “But they refrain from using contraceptives due to fear of their side effects.” -HCP9  “No one comes with a request for 12 years contraceptives, most of them come for 5 years contraceptives i.e., implants.” -HCP13  “But when discussing family planning methods, IUCD is not a favoured method. Nobody wants to put it in the uterus.” -HCP13  “The response of women was not so positive for PPIUCD.” -HCP14  “And the other thing, Depo-Provera and implants can work easily for a certain period once used, but IUCD is kept inside the uterus and in my understanding, clients may be scared and refuse to wear it because it is kept inside your sensitive part. I have had the opportunity to participate in many programs; there is a need of changing attitude towards IUCD.” -PP1  “People accept safe motherhood services as essential services whereas family planning services are neglected by the people and the system. Another thing is that if they have a problem of knowledge, they also have a problem of attitude.” -PP3  “…. But the attitude of educated people towards the use of contraceptives is not good. There may be some reasons why they refrain from using family planning measures. Reproductive health issues are still very sensitive.” -PP4  “Women are not so sensitive to their reproductive health overall. I feel they are less sensible about their health; maybe they think it a natural process.” -PP5  “There are fears of complications associated with all types of contraceptives. It could be IUCD, implant, injectables, or anything. No doubt, we need to work on that. People are not willing to keep something inside their uterus although it’s good.” -PP9  “There is fear of infection as well; Nepali women are afraid. One of the other factors is shyness. If you ask me to use PPIUCD, I will consider it inconvenient because I won’t keep something unnatural inside my body. Because you must go to the health centre and show everything (private parts), you must keep it inside, there is a feeling of shyness and discomfort, and I think there is less acceptance culturally, too. In our culture, it is still difficult to expose your genitals. Even though I am a healthcare worker, I feel inconvenient, and it seems like a major factor.” -PP10  “There are awareness programs, many kinds of programs are being done on behalf of NHEICC [National Health Education, Information and Communication Center] involving FCHV [female community health volunteers]. Even if they are informed against misperceptions, it is difficult to change the attitude.” -PP10  “People know what the side effects are when they eat Panipuri (a kind of street food); they can have diarrhoea, or jaundice, but people still eat it. But if you start talking about family planning, they start thinking of side effects, unwanted effects, complications, what they hear about it, and what happened to people, and they follow other people’s suggestions. They don’t feel family planning is important. Even when it comes to male vasectomy, they just make excuses saying their wife won’t let them do it. Even though the husbands don’t listen to their wife’s suggestions for other many things, they follow their wife’s suggestion at this matter.” -PP11  “The government of Nepal may advocate for PPIUCD, but since it must be placed in the uterus, women are initially hesitant. Who won’t hesitate to keep in private part!” -PP11  “As a clinician, no matter how many couples I met, it seems that the females are not interested in sex. Women usually say, “I don’t want it, sometimes it just happens”. I do not understand why it is not needed. One reason could be the probability of unwanted pregnancy in the absence of the use of contraceptives, so they might want to minimise the sexual relations as much as possible. That’s why there is a chance of conflict in the couple for which we are not able to do anything.” -PP11  “For postpartum family planning, we say we should start counselling from antenatal period, and we are also teaching the same. And... people know that a child will be born after being in a relationship, but people expect things won’t happen to them, which they do not want in their own case. But anything can happen to anyone.” -PP11  “And again, people question the use of family planning during the postpartum period saying the couple just had a baby. But sexual desire can come to a couple anytime, so they don't look at that part.” -PP11 |
| Social and cultural influences | “Whatever services they are getting are mostly influenced by the peers.” -HCP1  “In our context, their choice of contraceptives is mainly influenced by their peers rather than their family members. They opt for Depo-Provera because their peers are using the same.” -HCP2  “The main thing that influences their decision-making is neighbourhood. They say the neighbour’s daughter-in-law is using Depo, so they are also using Depo……They give examples of other women in their neighbourhood who suffered from the negative impacts of IUCD. Even if I counsel a lot, women don’t like to go for IUCD at the end.” -HCP4  “Women make decisions only after consultation with the husband, and even other family members. This is a big barrier women face. Even after the female makes her mind up and goes to receive service, she will be queried about her decision by her family members and neighbours.” -HCP5  “Although we say the female has the right over her body, there is an obligation to talk with the husband as well. We should start counselling from the beginning during antenatal care.” -HCP6  “According to what we understand, it seems that families play a major role in decision making regarding family planning or contraceptives. Some come mentally prepared to use PPIUCD by understanding its effect. Some get convinced to use PPIUCD when we counsel them in detail about its effects and side effects. Most of the women couldn’t decide to use PPIUCD due to family.” -HCP6  “In this context, I think it is a personal or individual decision ingrained due to tradition and culture. It feels like a negligence. It seems that even though the clients know everything, they don’t want to use it. This society is not like some Muslim community where family planning is restricted. Instead, they take abortion pills as much as they like; they don't even fear taking them, but they are afraid to use family planning methods and their side effects.” -HCP7    “If we change a woman’s perception about the PPIUCD, she will be convinced to at least try the PPIUCD, but importantly, if we can show that their friends, and neighbours are using it, there is chance of acceptance. We should provide them with success stories, and it can be effective.” -HCP7  “Family and husband have a lot of influence over their decision to use PPIUCD. Many clients come here to remove IUCD as their husbands complain of pain due to IUCD during sexual relationships…………………. Some people also complained of contraceptive failure as husbands threw the IUCD out during intercourse. Such things are not even reported in the research.” -HCP8  “Their selection of contraceptives depends on what their near colleagues and relatives have adopted. If they hear their friends have used Norplant/implant and are happy with it, they will also take it, and if it caused some side effects to their friends or if it does not suit them, then they will not accept to take it. Word of mouth from their friends has important effect on them.” -HCP9  “Sometimes women also come to discontinue the use because their husbands complain of the discomfort. They have reported pain due to Copper-T during the time of sexual intercourse” -HCP9  “Their family members object to their use of contraceptives, believing they won’t be conceiving child for 2-3 years even without the use of contraceptives…………. Even after counselling, if one of their family members provides their example of not conceiving a child until they menstruate, they won’t receive any contraceptives.” -HCP9  “The religious beliefs as well, especially in the Muslim community prevent them from using contraceptives. There is a huge number of people who believe that contraceptives should not be used. We can say we have that mass of population……………………. Although we say the female has the right over her body, there is an obligation to talk with the husband as well. We should start counselling from the beginning during antenatal care.” -HCP10  “They provide reasons for declining as their neighbours and friends informed them that Copper-t can pierce the uterus.” -HCP11  “We need to keep couples together for counselling……. I observed that the women’s choice of contraceptives is affected by the husband's preference. The wife does not decide anything until the husband agrees to the use of contraceptives.” -HCP11  “A woman’s family background also affects her decision to use contraceptives; if we ask her if she wants any contraceptives, she says, "I will ask my husband, I will consult at home."” -HCP12  “Then it also depends on what the mother-in-law says. This is what clients say, and we observe this practice in our practice.” -HCP13  “People have also heard of complications from friends and relatives. So, they didn’t have a very positive perception about it. It was really challenging to convince women in the first place.” -HCP14  “Family planning is still important, but women do not use family planning due to causes such as family limitation, husband and family support, and not knowing about family planning.” -PP3  “There could be other underlying factors at the community level that stops women, even educated women to use contraceptives.” -PP4  “One of the main things is that the barriers to taking family planning services can vary according to the community, women from different communities come to us to take the service. Especially, we perceived a big barrier in the Muslim community.” -PP5  “It is very rare that the husband or other family members bring women here to support them with family planning services. The users come here often for service, lying about going to market. Many report that they face criticism if their family members or husbands find out about it. This is a socio-cultural barrier. Besides that, women, themselves are not so sensitive to their reproductive health overall.” -PP5  “Due to cultural barriers, patriarchal society, family planning is considered women’s health issue and is the reason why women come secretly to get service." -PP5  “It also depends on how aware and supportive their family are.” -PP8  “The understanding between husband and wife, and role of in-laws affect their choice and reproductive behaviours.” -PP9  “My own niece got married at a young age. Her mother-in-law stopped her from taking oral contraceptives that she was taking. So, my niece had her first child when she was 17 years old. She also didn't allow spacing for the second child. If that happens in a well-educated family like ours, what will happen to the girls in the village?” -PP9  “One of the significant barriers is the lack of autonomy for decision-making. We share information as informed choices during the counselling time, but there are also situations where they cannot decide for themselves. First, they must cross hurdles to come to the service site itself. Even after they come for service, they need their family’s permission to make choices.” -PP11 |
| **Barrier theme 2: Postpartum intrauterine contraceptive device issues, preferences and competitive interest** | |
| Complexity of PPIUCD procedure | “Insertion of PPIUCD is a complex procedure and not all the healthcare providers are trained in PPIUCD. It needs a trained person to insert and remove this contraceptive. The easiest are pills and Depo-Provera.” -HCP2  “If you are trained, it can be inserted in one shot. The process of IUCD insertion is not super difficult. It involves assessment of the cervix and uterus and involves manipulation with a speculum, as the cervix is sensitive, it can cause discomfort. We measure the depth of the uterus.” -HCP4  “Applying PPIUCD is not difficult. It's not that difficult if we practice.” -HCP6  “If the healthcare providers understand the process, it is not a complicated process. Every nursing staff and doctor should learn this procedure. The chance of displacement is high in the involution phase, but again, if it is placed by a trained person with correct technique, there is less chance of displacement.” -HCP10  “Even after being a registered OB/GYN, those who have not inserted it before need training. Because sometimes, there can be complications during insertion, and usually, during insertion, you have to see which direction the instrument is to be inserted, and you have to know how many sponge holders will hold the cervix during insertion; otherwise, if they insert it without training, they might leave copper-t in the lower uterine section and may cause expulsion. It needs some technical skill. When we reach the fundus, we must come back 2-3 cm and release it. Even for the trained ones, if they are not practising, they also need refresher training.” -HCP12 |
| Health conditions, side effects and complications | “Its main drawback is the chance of expulsion. One of the side effects may be infection, but it can be cured.” -HCP1  “I have my own experience; I used to have heavy bleeding and abdominal pain while using PPIUCD. I conceived baby even while I was using IUCD.” -HCP4  “It is very effective, but there is also a risk/hazard if the insertion technique is not good, such as high expulsion risk. Sometimes, even if you are a trained person, there may be chance of perforation, depending on the shape and size of the uterus, sometimes there can be complications.” -HCP9  “I have seen such complications when a patient came for the removal of the PPIUCD. The Copper-T had pierced the bladder wall.” -HCP10  “Many women report vaginal discharge, lower abdominal pain, backache, they don’t like to use IUCD.” -HCP4  “I have seen complications in two cases….……. After looking at the x-ray, it was found that the IUCD was upside down.” -HCP7  “A PPIUCD is somewhat different; placing an IUCD during postpartum phase has a slightly higher chance of expulsion.” -HCP7  “One woman became pregnant even when she was using IUCD and had to perform a medical abortion. There are many who come with complaints of discomfort” -HCP8  “There will be some irregularities in the two or three menstruation cycle in the beginning after the use of contraceptives; it won’t be like the normal menstruation cycle. They don’t believe if we say that this is normal process after the use of contraceptives.” -HCP10  “PPIUCD can be provided successfully but there is a high chance of expulsion, but we call customers after a month for a follow-up.” -HCP11  “Some of them come with excessive bleeding due to menorrhagia, or metrorrhagia, and request to take them out. A few side effects are sometimes expulsion, sometimes thread coming out and in very rare cases uterine perforation.” -HCP12  “Some 1-2 people out of 100 come with complain of irregular menstrual bleeding and heavy bleeding. We always tell them these side-effects and we are saying that these side-effects are normal to some extent and may get better with time.” -HCP13  “One of the important challenges is their own health condition. The first thing is to look at their own history, whether they have any pre-existing disease or not, and some other past histories. We need to look into past histories. Usually, patients with normal vaginal delivery don’t have disease history. It will be known if someone has a history. And the main things to be decided by the history are whether they have a history of PID [Pelvic Inflammatory Diseases], repeated infections. All that should be taken into consideration, and we can group patients according to their disease conditions or history before delivery.” -HCP8  “It is kept in the arms, they don’t need to expose their private parts [vagina], they don’t need to maintain hygiene, so most women use Implant in this district. Even when I pursue women to use copper-t, they decline saying it causes vaginal discharge. It is not even preferred by educated people.” -HCP4  “We once had a funded project at around 2008 where we focussed on increasing acceptance of IUCD, and we worked for about a year. Under that project, we counselled and motivated patients and provided some snacks and drinks for the ones inserting IUCD, many also opted for it, but many clients came back to remove it complaining discomfort, bleeding.” -PP5 |
| Preferred alternatives | “Women hesitate to go for permanent contraceptives such as Mini-lap. Women usually prefer Norplant and Depo-Provera.” -HCP1  “I personally like it. But it is very difficult to get patients’ acceptance for this contraceptive. implant is preferred among postpartum family planning contraceptives.” -HCP7  “We only get about 2,3 cases in a year and Implant is popular compared to IUCD in our facility.” -HCP2  “In our area the most preferred contraceptive is Depo-Provera followed by the implant. Nowadays, we don’t find clients taking OCPs for a long time. Previously, women were also found to be using OCPs for a long time up to 10 years.” -HCP2  “Women in this area prefer Depo-Provera and implant. Only some women use Copper-T.” -HCP3  “But most of the women deny IUCD so we focus on implant as we can’t give pills to a breastfeeding woman. That is why we are focussing on implants.” -HCP3  “Women prefer implant, they even opt for frequent insertion of implant. I have even kept implant for up to four times in some women again and again. Even the PPIUCD users who have used previously came to remove it and shift to other kind of contraceptives” -HCP4  “It is kept in the arms, they don’t need to expose their private parts [vagina], they don’t need to maintain hygiene, so most women use Implant in ____ [a district]. Even when I pursue women to use copper-t, they decline saying it causes vaginal discharge. It is not even preferred by educated people.” -HCP4  “However, who are with their husbands, they use contraceptives, but Copper-T is not the preferred method of contraception.” -HCP4  “Pills, depo, Norplant are the preferred contraceptives by women.” -HCP6  “Many women are using implants because they are placed in the arms.” -HCP9  “Most of the women use Implant and Depo-Provera.” -HCP13  “In our area the most preferred contraceptive is Depo-Provera followed by the implant. Nowadays, we don’t find clients taking OCPs for a long time. Previously, women were also found to be using OCPs for a long time up to 10 years.” -HCP14 |
| Beliefs about consequences | “Sometimes they worry that they can’t find the threads of IUCD once placed, it can only be found with ultrasound.” -HCP3  “I have also heard of uterine wall perforation when untrained personnel tried this process.” -HCP4  “Earlier, a couple of people had complained that they had some discomfort with the thread of copper-t.” -HCP6  “I have seen complications in two cases. While working in a maternity hospital, a patient abused me very badly. There was a patient willing to use PPIUCD, the counselling was well received, we took consent of both the patient and the visitor. We had a trainee student, so I allowed her to insert the PPIUCD under my supervision and the patient was then discharged. After some time, they came back, now I don't know the exact duration, they came with an x-ray in their hand and insulted me by saying, the IUCD moved upside down. The patient scolded me for forcing her to use it while she was unconscious. she was not convinced that it was only inserted with their consent. After looking at the x-ray, it was found that the IUCD was upside down. Later the doctor took it out. I have seen so many cases, but I do not know how it got upside down.” -HCP7  “It is very effective, but there is also a risk/hazard if the insertion technique is not good, such as high expulsion risk. Sometimes, even if you are a trained person, there may be chance of perforation, depending on the shape and size of the uterus, sometimes there can be complications.” -HCP9  “I haven’t encountered any complications at the time of insertion of PPIUCD when I was doing it, but I have seen such complication when patient came for the removal of the PPIUCD. The Copper-T had pierced the bladder wall. That's when I realised that the Copper-T is also dangerous. There was also a case of abdominal wall perforation. Such complications happen.” -HCP10  “There is about a 10 to 20% expulsion rate. But high expulsion rate doesn't mean you shouldn’t use it.” -HCP11  “Expulsion rate is not very high but higher compared to non-postpartum mothers, we found about 2-3 expulsions out of 100. But it is not because of expulsion rate we stopped putting PPIUCD.” -HCP12  “Another major setback we realised is the complication that we faced. Normally, when we insert copper-t in non-postpartum women, we cut the thread and leave 2-3 cm of thread, but we need to keep the thread of copper-t as it is when we insert this in postpartum women, which is quite longer. We counselled them that the thread should be visible after about 6 weeks when the uterus is almost the normal size. Many patients came back as the thread was not visible at follow-up time. When we performed the ultrasound to examine if the copper-t was not misplaced, the copper-t was in place, but the thread was hidden. We found the thread itself kinked during the involution in some women. Due to which we experienced reluctance from patients later. Many people opted to take it out due to this reason.” -HCP12  “I had instances when I had to leave OPDs to take patients to the ward where the ultrasound machine was, but it was not feasible to do every time and when we send them to central radiology department for ultrasound…….” -HCP12  “I have not performed USG on IUCD users for finding the lost IUCD thread, but I have heard about such cases; there was a case with loss of IUCD sheath.” -HCP13 |
| More urgent care takes priority | “We equally prioritise, but the essential thing during the time of pregnancy health care is pregnancy health because, at that time, the woman has two people in her body. And if anything goes wrong, it can cost two lives simultaneously. That’s why maternity care is a priority. Family planning is also a priority, but the health of pregnant women is of central importance at that time. When a woman is in the later phase of labour when they are serious, we need to focus on delivering her; otherwise, it is risky to the woman and the child. Because the mother had carried the baby for 9 months, if the mother is saved and the baby suffers complications or vice versa, it is a huge challenge, and their immediate medical care remains the utmost priority.” -HCP4  “We focus about delivering babies quickly and giving time to another patient in waiting. So, when we ask them if they want the PPIUCD, if they say they are willing to have PPIUCD, we provide them, but we don’t have time to provide them reassurance………But we prioritise on other outcomes of childbirth. Because it is not like the patients will have complications if PPIUCD is not given, but if you focus too much on the IUCD in one case and cannot go to the other patient, there can be complication in another case.” -HCP7  “It cannot be said that it is not a priority of the government, but its implementation is not very good. But as we have seen, as government doctor and authority at the government office, I have seen that everything is being done, but there are some weaknesses. Otherwise, why would family planning programs fail?” -HCP8  “To say the truth, postpartum family planning counselling does not get enough priority.” -HCP9  “If we look at our practice, we are not putting enough effort and not prioritising enough.” -HCP10  “Hospital leaders should be focussing on all this thing. What we are currently focussing on management of complication of pregnancies, maternal problems, newborn problems, lot of other issues but family planning need also must be given priority.” -PP9  “We are more concerned about the obstetric complications than postpartum counselling.” -HCP10 |
| **Barrier theme 3: Inadequate capacity and capability** | |
| Inadequate staff and high workload | “We have a high workload. One of the staff trained in the PPIUCD just left. There is a high turnover of staff. We can’t stop them from leaving as well.” – HCP3  “The truth is the staff is low currently. There is a rush in the antenatal, postnatal, labour and gynaecology wards during duty hours. Sometimes two staff members also must handle one shift.” -HCP6  “As doctors deal with many complicated conditions, the doctor does not have time to do family planning counselling. However, nurses working in the maternity ward are trained with SBA training in all government hospitals, health posts, and even the private sector. They are the ones responsible for implementing the PPIUCD.” -HCP7  “I am very unsatisfied with the doctor-patient ratio because if we talk only about today. I checked/looked after 70 new cases and 30 follow-up cases visiting the outpatient department. I had to look after them all by myself. Just by looking at this number, you can understand the problem of the doctor-patient ratio. We don’t have time to look at everything in detail. We don’t have time to maintain the quality of care because there is no time to look at each and everything, that's why the doctor-patient ratio is a big problem, the nurse-patient ratio is also very bad.” -HCP8  “At the moment, there are only two staff members in the maternity ward. When one staff conducts delivery, we need another one to receive the newborn.” -HCP9  “Similarly, we have few staff, there are three nurses in one shift and doctors are busy with their own operative and post-operative cases, complicated cases with no time to counsel patients comprehensively.” -HCP10  “I recently worked in the busiest central maternity hospital in Nepal. Pregnant women were seen by the residents and consultants, and we had to attend in even up to 1000 women a day in total.” -HCP10  “There is a huge difference in treating educated and economically able people and dealing with a very marginalised population. Even for a normal procedure, it takes us about 1 or more than 2 hours to make them understand that they are having a normal delivery or caesarean section. This is too time consuming otherwise the workload is alright here.” -HCP10  “The reason why antenatal counselling is not successful is that we have not set a limit on how many patients can be seen in a day, and we are not able to set a limit now. For example, if you have 5 providers and we don’t consider how many patients each can see in a day by allocating 15 minutes per patient, we may end up scheduling far too many patients. We crunch as many patients as possible we can, and there is no way to see so many patients in a day. This results in a lack of proper counselling.” -HCP11  “Many nurses in the labour room have changed, many nurses who have taken training have been shifted to other wards, and many have already gone abroad. But the remaining trained ones can provide this service if practised once or twice or watch the video.” -HCP12  “Overall, we see 300 patients per day on average in the outpatient settings. 5-10 minutes can be given to a patient. It is manageable.” -HCP12  “The status of human resources is insufficient at health facilities where there are 15-20 thousand deliveries per year………. Tertiary level hospitals like ours are being overused.” -HCP14  “Some of the facilities at peripheral level are being underused and tertiary level hospitals like ours are being overused.” -HCP14  “In private health facilities, there is frequent staff turnover.” -PP1  “After federalisation, there are not many permanent health workers, especially in hilly areas because there is no permanent workforce; they are temporary or on contract, and if we give some training, after sometimes, the employee leaves and goes to another place and another new employee comes completely blank.” -PP2    “Our eight facilities don’t have all these services, and it was because of shortage of trained human resources…………………………. We also don’t have gynaecologists at basic public health facilities, prompting us to visit gynaecologists at private health centres. Gynaecologists won’t do anything about RH [reproductive health] in private health facilities.” -PP4  “In addition, it seems that the health system should be strengthened, especially after the federal system, there is no staff position at various level of health facilities.” -PP5  “The major provider of family planning is the nursing staff in our country, and we are now calling it midwifery. Because the same nurse must work everywhere, the counselling is not effectively delivered” -PP6  “In the case of PPIUCD, especially in the high delivery volume sites, there is a lack of human resources. We should invest on training skilled human resources.” -PP7  “If we look at the current scenario, the nurse-patient ratio is not satisfactory; midwives are even still not available. Based on the nurse-patient ratio, it is low not only in the maternity clinic but also in other general wards. But we are not able to implement according to the guidelines, but we are attempting continuously to implement accordingly.” -PP8  “The community health workers, such as female community health volunteers, are also almost non-functional in urban and peri-urban areas. They are working actively only in remote areas.” -PP12  “I haven’t seen any community volunteers proactively doing house-to-house counselling in urban and semi-urban areas. In remote areas, where mass education is usually provided during a mass campaign, we don’t have community healthcare workers for community outreach.” -PP12  “Be it [a teaching hospital] or the maternity hospital, or any big hospitals with high obstetric caseloads, they already have a resource crunch, we don’t have a mechanism to send additional staff there, and whatever staff they have, they also get rotated in the departments. Scale-up and implementation are only possible if they are done intensively as a dedicated program. Otherwise, the piecemeal approach amidst limited resources, although we are trying to push forward, is not sufficient.” -PP12  “Another aspect is a high turnover of trained staff. Trained staff also change their workstation and work in other sectors as well. Sometimes there are trained temporary staff leave for foreign employments, and it is normal to go to places, where there is a better opportunity” -PP12 |
| Inadequate training | “We just have one nurse who is trained in the PPIUCD, and in her absence, the service is interrupted. There is only one district hospital in such a large geography. We have many rural municipalities to be served by this hospital, but about half of the people don’t have access to this hospital because of the distance.” -HCP2  “Even though the government have invested in the public health side, the government have focussed less on the clinical skills of healthcare providers if we look at the health spending pattern.” -HCP2  “Family planning counselling and provision is important…………………………. We also need counselling training.” -HCP3  “Previously there used to be 14-day training especially family planning counselling for all staff but we do not having such training these days. As a result, we are providing only contraceptives that women ask for in general.” -HCP4  “There is a training package in Nepal called COFP [comprehensive family planning counselling], which is taken by a few nursing staff. I can't say exactly how many have taken. We have trained a few doctors in Mini-lap, and there is a few nursing staff trained for implant and IUCD, but not so many. There are one or two trained providers in our maternity unit; others have not taken it. Those who have been trained only work in the family planning section.” -HCP5  “In addition, we lack trained staff to deliver services. It seems to me that we don’t have enough training frequently.” -HCP6  “At the moment, we do not have separate training for counselling on family planning. The counselling training is given as a complementary to the PPIUCD insertion training.” -HCP6  “Since the skill is not used regularly, a refreshers training package is needed as skills are forgotten.” -HCP7  “In particular, there is only a little introduction in SBA [Skilled Birth Attendant] training. Otherwise, it is only as much as what is read in the college course, and we learn only by observing how our seniors are doing it, otherwise we do not have extra knowledge as separate family planning counselling, and the counselling skill is not practical in the course book.” -HCP7  “Most of the maternity staff are trained. but I don't know exactly whether their training is specifically for family planning or PPIUCD, but they are trained staff of family planning.” -HCP8  “Personally, I have not received any training, but the healthcare workers of different levels have training, like the nursing staff who are with us. As a gynaecologist, I already have such skills. We have nursing staff who have received training, and they also provide services that I have not covered. -HCP8  “The basic skills of the staff who have taken SBA training are evaluated and coached to see in every 6 months; it is not possible to do it for everyone; staff are sent for training whenever the government asks.” -HCP9  “Every nursing staff and doctor should learn this procedure. It's our duty and even those who have not received skilled birth attendants training, often conduct delivery. Therefore, I think it would be better for them to receive training as well.” -HCP10  “But other times we are busy with caesarean section, hysterectomy, complicated vaginal delivery. That's why nursing staff should be trained well for this skill.” -HCP10  “All colleagues are skilful, and special training has been given to all of them. All of them have taken SBA [skilled birth attendant] training. SBA also has this component of PPIUCD, and separate training is also given. Doctors now don’t need ASBA [advanced skilled birth attendants] training, but nurses need training.” -HCP11  “Even many gynaecologists do not know about PPIUCD, especially the practicality of this procedure. Theoretically, they may know. Many of the colleagues who took this training have already left the institute. Yet, there are about 6-7 remaining people who are trained. There is no monitoring as well. Many nurses in the labour room have changed, many nurses who have taken training have been shifted to other wards, and many have already gone abroad. But the remaining trained ones can provide this service if practised once or twice or watch training videos.” -HCP12  “But there should be some strategic changes in how we train healthcare providers. For example, the medical officers posted in the local level hospitals, such as Dhankuta, Inaruwa, Rangeli, who serve there to accomplish their mandatory 2-year service after medical graduation, are trained. Maybe they are now studying surgery, ENT, GI surgery, or psychiatry, and if so, they won’t continue providing PPIUCD. There is a necessity to decide who to train as training also means a financial burden.” -HCP12  “All of us don’t have PPIUCD and implant training, but it is our custom to refer to that service wherever it is available. We have a good referral system.” -HCP13  “In our country, we have restricted practice for the professionals with training certificates to provide any services, but I think a training certificate is not needed for this service. We don’t require 2-3 days of training; rather than training, there should be someone, a supervisor, who can oversee the procedure and guide professionals in the beginning. There are certain steps to follow, and when they follow those procedural steps, a supervisor should guide them. Otherwise, this procedure should be included as an essential skill for the requirements of the MD program, similar to ANM, staff nurse, B.Sc. Nursing. And such procedure can be done under the supervision of the supervisors in the ward, in charge.” -HCP14  “The counselling rate was higher when the counsellors were hired. Counsellors do not continue now. The challenge is human resources in our settings. Now there is no continuation of counsellors.” -HCP14  “In addition, in some cases, the trained staff can also have transfers within 2 years. Regarding training, we don’t have skilled human resources to provide training, and the authority to conduct training but we have many untrained staff and limited staff get training. It is also problematic.” -PP4  “We cannot train ourselves; the NHTC [national health training centre] has not called even one person for training.” -PP4  “Unfortunately, training is provided by the national health training centre and provincial health training centre. For example, this year, the training centres allocated two spots for IUCD training in our municipality. At this rate, it will take years to train all our staff and to have health facilities with all the contraceptive facilities when the training centres provide training to only 2 staff a year. We have already assessed and identified who needs training, but unfortunately, we can’t organise training, and the municipality doesn’t have that level of human resources and facilities to be a training centre.” -PP4  “When there is availability of devices, there are no trained human resources, and when there are trained human resources and devices, counselling has not reached adequately yet.” -PP6  “And if we look at the number of trained providers, the proportion of IUCD training is very low. But the proportion of IUCD training is very low, it is less than 25%. That creates a gap. We need to focus on both demand and supply…. But they don't even demand IUCD training much.” -PP7  “It is said that basic health service should be in basic health facilities, but there is an extent of access to training, and depend on how much the government can provide.” -PP8  “If you ask health workers, Gynaecologists, and nursing staff in various forums, they do not have accurate information about it. There is no knowledge of what to give from when the day is due, and there is no information about what can be prescribed next time.” -PP12 |
| Variability in family planning counselling provided | “If they are concerned, counselling is done, but we give them contraception counselling usually to those who come interested in family planning. If you are not having a baby even after trying for many years, or if you want to do birth spacing, then if you want to have a baby, you will be counselled, but not during antenatal care and when there is a postnatal phase, then we do counselling……………………….. It varies by gravida. If the mother has gravida of 6, 7, or 8, we try to convince them to opt for contraceptives as much as possible during antenatal care because the higher gravida increases the risk of obstetric complications and maternal deaths.” -HCP1  “It requires proper counselling to make clients understand about postpartum counselling. Maybe We haven’t been able to provide enough time for counselling.” -HCP2  “We counsel everyone, especially when women don’t want to have children, we counsel them for permanent sterilisation, and when they deny permanent, we provide them the option of long-acting reversible contraceptives.” -HCP3  “Usually, we provide them short-acting if this is their first child as they need to space for only two years, but if they have two children already and they don’t want any more children, permanent sterilisation but if they need more children, we suggest short-acting.” -HCP4  “If they express that using contraceptives is essential to prevent further childbearing and request immediately after delivery, there is PPIUCD, which can be kept internally in the uterus and works for 12 years.” -HCP4  “Doctors also ask women about the number of children they want to have, they may not be able to dedicate a long time for counselling and may not follow appropriate methods of counselling, but the attending doctors ask women if they want to do mini lap along with caesarean section.” -HCP4  “If a separate staff is kept for counselling only, even if it is not possible in each shift if it is kept like 8 am in the morning to 5 pm in the afternoon, it would be best…” -HCP6  “As doctors deal with many complicated conditions, the doctor does not have time to do family planning counselling. However, nurses working in the maternity ward are trained with SBA training in all government hospitals, health posts, and even the private sector. They are the ones responsible for implementing the PPIUCD.” -HCP7  “We focus on delivering babies quickly and giving time to another patient in waiting. So, when we ask mothers if they want the PPIUCD service, we provide it if they express their willingness. But we lack time to offer them reassurances they need.” -HCP7  “In such cases, we do not have enough time to counsel patients who are waiting in the antenatal ward to deliver the baby. We miss that window of opportunity. Counselling is harder once they start having labour pain. So, it is better to have a separate dedicated counsellor.” -HCP 9  “Integration of family planning and maternity care also gets some space, but it is not possible to follow the cafeteria approach of contraceptives during antenatal care. We counsel on a case-by-case basis; for example, if a woman comes for antenatal care with a history of caesarean section, we ask patients for BTL [bilateral tubal ligation]. We hardly talk about PPIUCD or other family planning methods at that time. We are so much into scissors and scalpels. We are a little bit biased.” -HCP10  “There is not enough counselling during antenatal because all cases are not booked cases. The nurses meet them at the time of labour when women are admitted for childbirth. They receive patients at labour or during some complication, and I don't think anyone wants postpartum family planning counselling at that time. There is a lack of connection or bridging or integration.” -HCP10  “I won’t say we can counsel every woman in the outpatient department during antenatal visits as we see about 200-300 clients in a day, so I won't say we can counsel everyone, but at least we can counsel 10 of them with important indications such as gravida more than 3.” -HCP10  “On top of it, there can be significant hormonal imbalances during postpartum phase e.g. abnormalities like abnormal bleeding, increasing cases of adenomyosis. We also automatically suggest pills [oral contraceptive pills] because it is therapeutic too, it is like a preventive measure, so it is a friendly approach. If women have multigravida of 6, or 7 or more, we recommend them PPIUCD, and when clients don't have any other options, we recommend PPIUCD.” -HCP10  “We provide counselling especially to each case of caesarean section and multigravida women.” -HCP11  “If clients come to us saying they want to use PPIUCD themselves or if they want to use some family planning measures, then we suggest using suitable family planning measures according to comorbidities, age etc. As such, they have a history of multiple parity or elderly multigravida, we suggest they to use family planning measures, be it permanent, long-term contraceptives. But we do not do counselling for those who have one or two children in the OPD [outpatient department]. If we are to perform A caesarean section, we always give them the option of permanent sterilisation. If clients want more information about the contraceptives, we send them the nurses for more detailed information and service.” -HCP12  “Some of the facilities at the peripheral level are underused and tertiary level hospitals like ours are overused. If we can maintain the balance, we would have got enough time for patients, and we would provide them counselling using the cafeteria approach.” -HCP14  “Counselling is not good in our practice in Nepal. According to the ones who observe it, who see it, who experience it, if we must rank the quality of counselling according to their experience, then it would be 3 or 4 out of 10.” -PP6  “Another challenge is the availability of trained human resources. If you have reviewed published documents, the quality of counselling provided to women and girls is compromised.” -PP7  “If you counsel women properly, they accept IUCD, but the procedures and its benefits are not properly explained to them. If women are just explained saying this is an external thing, IUCD will be inserted in their uterus, when women already have misconceptions such as it pierces through the uterus and reaches the heart, people can die, it causes cancer, and so on, the women won’t just accept it.” -PP7  “In 2010, we brought out a booklet called “Misconceptions about Family Planning” through research and understanding of the community in 20-21 districts. It contains misconceptions and information about IUCD. Rather than misconception itself, it is due to a lack of true information and poor counselling that women are refraining from using IUCD and we can overcome this misconception through quality counselling.” –-PP7 |
| Attitude of healthcare providers | “The staff should also be motivated and should commit to providing this service. If the staff themselves are skilled and come forward, the uptake can be increased.” -PP1  “There may be demotivated staff, some colleagues have some political pressure from the union, there are many kinds of pressure on the employees, because of those reasons, it can’t be said if all colleagues are putting their best effort.” -PP2  “There may have been barriers like service provider's performance and attitude, but such things have not come to the notice of the hospital.” -PP3  “Those with poor access who come to our basic health facilities will get fundamental/basic services, and the poor people will be neglected due to subjective behaviours of the providers. No matter how much we talk about equity, this is a human tendency; the treatment will be different. Therefore, unless educated, respectable population do not come to our urban health facilities, the service standard of our health facilities won’t be very good.” -PP4  “If I must take my wife for delivery/ANC, I will take her to the place where there is a gynaecologist. Does a person living in Kathmandu go see a gynaecologist/obstetrician or go see an ANM? As soon as you go to the gynaecologist, who are instantly available in the private health facilities, they won’t do anything about RH [reproductive health] in private.” -PP4  “However, on regular days, hardly anyone talks about family planning during the ANC. When women come to ANC, the providers check them and give them iron pills, folic acids, and calcium tablets. Providers don't seem to talk much about family planning.” -PP7  “If the stakeholders focus a little on PPIUCD and prioritise it in their service, as I said earlier, clients may opt for it. But they don't even demand IUCD training much. They mainly prioritise implant training and SBA training. Although IUCD is also embedded in SBA training, they are not providing service.” -PP7  “But we also observed and perceived the provider's bias when we talked to the healthcare provider; they usually refrained from putting IUCD, saying they have poor personal hygiene. That kind of bias is prevalent in the providers. I think first we only talk about behaviour change of the users, but if we look at, particularly for IUCD, providers also need to change their behaviours.” -PP7  “Practically, it is not difficult to manage the space as well. You can simply use a curtain as a divider to provide privacy to patients. I used to work as a district officer in a remote district; I had to walk for about 2 hours to reach the basic health centre; there was no motor road, and the health centre had two rooms, but it was being managed very well. In one room, he used to keep the equipment and check the patients in another room. Especially when someone came for family planning or ANC, the provider used to provide service by putting a curtain in the storeroom. Not only that, but the provider also requested IUCD training, and he was providing IUCD services to rural women. When the facilities are good, good service should be provided, but when the health facilities don’t have adequate infrastructure, we need to find alternatives. There is no excuse for not being able to provide the service because in the current perspective of Nepal.” -PP7  “In a recent meeting, the director-level position was called for participation in the meeting, but they sent another staff as their replacement; that staff does not have the capacity of that level. The replacement will share details with his director, but will that be effective?” -PP9  “Many young doctors are not interested to learn the family planning techniques first because their focus is diverted to more advanced training such as in vitro fertilisation and laparoscopic surgery technology, but not to small things. This is just my general observation.” -PP9  “If we talk to healthcare providers now, they say we don't have free time, but the counselling is part of antenatal care; we don't need to tell a long story for providing family planning counselling. You can simply assess if they have a plan for spacing, long-term spacing, short-term spacing or no spacing at all. Is it too much to ask four or five questions? Counselling can be done even while doing other things…... However, we shouldn’t coerce clients.” -PP9  “There are also issues with service provider’s attitude, or with the leader’s attitude. We can see all those things if we analyse them minutely. In today's digital age, I think the biggest problem is attitude. We try to stay in the same comfort zone and give ourselves excuse saying we have not received the training; we have not received the guideline books.” -PP10  “We should also counsel healthcare providers for providing counselling because, as a provider as well, I am not saying all providers are doing very good counselling and involved in the patient’s care. I have seen the gap from the provider side as well. In Nepal, a kind of discipline is lacking in all areas, maybe because the system of quality assurance is not so strong, political instability, or a highly politicised system. Discipline is a very lacking area in Nepal.” -PP11  “Now if you look at the health post, from HA [health assistants] to AHW [auxiliary health workers], ANM [auxiliary nurse midwives], staff nurse, there are four cadres as our mid-level provider, they all get training in family planning, they all get training, but how much services they are providing depends on the provider’s attitude, determination and tendency.” -PP11  “But here in the urban area, there are even bigger challenges; family planning services are not available in big hospitals, and the other important thing is that obstetricians do not prioritise family planning services. It’s not a lucrative professional focus for them.” -PP12 |
| **Barrier theme 4: Inadequate Investment and priority** | |
| Limited resourcing or preparedness of health facilities | “Looking at this, it seems that the contraceptive service during postpartum is a little better now compared to before, but it does not seem to be as much as it should be. It might be due to lack of resources, maybe not enough resources.” -HCP6  “Counselling is impractical once they start having labour pain. So, it is better to have a separate dedicated counsellor. Some budget can be allocated for this.” -HCP9  “We also need to create a dedicated space for daily counselling. What we don't have is adequate human resources, time and a system to motivate them.” -HCP9  “We can do small things. We can keep a small section about contraceptive/family planning/abortion in the antenatal card so that when we must fill that card, we can ask them about it while filling in the card.” -HCP10  “However, when we provide them counselling during the doctor’s round where other patients are also present. So, if one person makes a particular choice, everyone around starts making the same choice. If one person first denies, then everyone starts denying. If the first one says ‘yes’ and the second one says yes, more people follow the same choice. There are 5-6 people in one room in antenatal care waiting for the childbirth. Antenatal care outpatient services are provided by the doctors in the outpatient department setting in this hospital.” -HCP11  “Earlier, _________ [national professional society] had employed two nurses as counsellors during the program, but later the counsellors were removed after the funding was over.” -HCP11  “We have no separate counselling room or separate insertion room or separate designated human resources for PPIUCD.” -HCP12  “One big barrier is the lack of ultrasound machines in the OPD to assess the complications. I have had instances when I had to leave OPDs to take patients to the ward where the ultrasound machine was, but it was not feasible to do every time. We also refer to the radiology department for ultrasound.” -HCP12  “One important thing is that clients come here for delivery from different distant places. But once the insertion is done, a big question arises: Where should they go for follow-up? There is no provision that the government designated specific places to go for follow-up later. When clients visit their local places with problems such as loss of copper-t thread or when it is not visible, they are usually turned down or asked them to visit the places where it has been inserted. It poses a big financial burden for travel and medical procedure.” -HCP12  “Some of the facilities at the peripheral level are being underused, and tertiary level hospitals like ours are being overused. If we can maintain the balance, we would have got enough time for patients and we would provide them counselling using the cafeteria approach.” -HCP14  “Now what we need is resources to move forward programmatically; we need human resources; what we have is not enough. This is not an excuse; it is a fact.” -PP12  “The biggest challenge we faced was the lack of information about the service and technical capacity.” -PP12  “In our municipality, there are 8-10 nurses who have received IUCD and implant training. However, there are no birthing facilities in all basic health facilities, and the trained nurses are not exposed in the practice. As IUCD is not popular, we have encountered the problem that the trained professionals start forgetting the skills and knowledge.” -PP1  “Another thing is that we also need good infrastructure. But we are providing services by renting two rooms in some places; we don’t have our own buildings for basic health facilities in each of the 19 wards. Sometimes, when commodities are out of stock in some health facilities, we bring them from internally over-stocked health facilities.” -PP1  “Now the local municipality can do the small things, the basic things and the primary approach. But it would be faster and easier if there was a little support to operate special services that require special human resource development.” -PP2  “But only 2 out of 10 health facilities in our municipality have all these family planning commodities including IUCD.” -PP4  “We also don’t have gynaecologists at basic public health facilities prompting us to visit gynaecologists to private health centre. Even now, the maternal services are provided by the auxiliary nursing midwife in our health facilities. If I must take my wife for delivery/ANC, I will take her to the place where there is a gynaecologist. Does a person living in Kathmandu go see a gynaecologist/obstetrician or go see an ANM? As soon as you go to the gynaecologist, who are instantly available in the private health facilities, won’t do anything about RH [reproductive health] in private.” -PP4  “Our facilities run in 2-3 rooms of a building, and this is a major setback for our population. RH is a sensitive issue; privacy and confidentiality issues are very important. They are not interested in coming to the health facility running in 2 rooms and taking RH service. Normally, people don't like such provision…. First, the physical infrastructure should be convincing enough to make them enter through the door of the health facilities…. I have also experienced that physical infrastructure plays a big role as the providers need to have a proper and adequate place to provide services. Otherwise, the training will be a waste.” -PP4  “Another challenge is the availability of trained human resources. If you have reviewed published documents, the quality of counselling provided to women and girls is compromised. For example, if you go to the _______ [a zonal hospital] and see the maternity ward, you can see women sitting on the floor of the maternity ward. But looking at it now, it seems that the initiative has gone nowhere.” -PP7  “If we look at the long-acting family planning measures, only around 50% of health facilities have long-acting family planning services………. The challenge is if anyone demands long-acting family planning methods, reversible or permanent, they must wait, there is no easy access.”-PP7  If you look at the strategy/policy document, this is part of basic health service and should be available in all basic healthcare centres (basic healthcare service centres), but not all BHSCs have this provision. If we assess the situation of safe abortion care services, less than 1 in 5 peripheral public health facilities have functional, safe abortion service. The challenge is if a man or woman demands long-acting family planning methods, reversible or permanent, they must wait; there is no easy access. -PP7  “Not having dedicated clinics for family planning is the biggest challenge and the most challenging part is the urban and semi-urban areas. In rural areas, people go to the health post, where this service is available.” -PP12 |
| Issues with oversight | “We don’t have particular supervision mechanism for postpartum family planning, but skilled birth attendants supervise nurses when required” -HCP1  “But as family planning is not directly related to mortality, that’s why it doesn’t have special review programmes.” -HCP6  “At the hospital level, we don’t have any review program; we have a monthly report presentation but there are no such reviews.” -HCP7  “We don’t have anything like need assessment for staff.” -HCP8  “PPIUCD is a part of the SBA training, and once you are SBA trained, there is no supervision, no motivation and no perks. We have never been asked how many PPIUCDs have we kept. Although we report the use of family planning, we have never discussed about what should be the minimum number that should be gone, why it is less utilised or what made the utilisation more in some months, and we have never focussed on the particular contraceptive such as PPIUCD only.” -HCP9  “It is possible to do mass counselling in the OPD but needs the involvement of authority to divide and designate tasks among the providers. There should be clarity about who is going to do what. The head of the department should actively divide roles and responsibilities.” -HCP12  “There is no monitoring for this service…………. The first thing is that a strong initiative should come from the government. Health facilities may start by corresponding and collaborating with government agency like Ministry of Health and Population or province-level ministries; otherwise, continuation is difficult. If the government wants to continue, then it will continue. Otherwise, the program will run until there are incentives.” -HCP12  “Specifically, there is an issue of who will do the monitoring for postpartum family planning. That's why I don't see that much monitoring now; before there was a lot of interest during the intervention phase, and when federalism was not effective, there was interest from the centre.” -HCP14  “From time to time, we used to get a visit from a professor from the UK, but not anymore while PPIUCD was implemented first.” -HCP14  “We have separate indicators for family planning and safe motherhood, but the current information system and reporting system don't seem to have indicators to measure how integrated family planning services and maternity care are. A major indicator in family planning is CPR [contraceptive prevalence rate]. The CPR was at a very constant level in the last 4-5 years in our district, it was stagnant at around 29, 30, 31, now it has increased to the level of 40.” -PP2  “There is nothing we can do without personal relationships to get a job done in Nepal. It wouldn’t have been necessary to have a personal connection if things could be done through a system. After using all personal connection and leverage we organised Implant and IUCD trainings for staff.” -PP3  “The allocation of budget for health sector at local level depends on how much we can convince the administrative executives.”-PP3  “Now, a person I personally know has joined the provincial government. So it is easy for me to get approval. There is nothing we can do without personal access to get a job done in Nepal. It wouldn’t have been necessary to have a personal connection if things could be done through a system. After using all personal connection and leverage, we organised Implant and IUCD trainings for staff.” -PP3  “The government policies and programs don’t penetrate the practice of private health facilities. They don't talk about family planning.” -PP4  “There is no reproductive healthcare coordinating committee at the municipality level, nor are there some guidelines.” -PP4  “Earlier, the district had a dedicated focal person for specific services. For example, for RH [reproductive health] programmes, there used to be a single focal person, whose job was focussed on RH [reproductive health]. For example, I didn't work in other sectors except in the RH [Reproductive Health]. We had a direct link with the family health division, and we used to implement programmes following the direction of the family health division. There used to be monitoring and time to time feedback mechanism.” -PP4  “At the moment, no one knows what the chain of command is. ……………………. But the decision-makers at the local level are the administrators and elected members who need explanations.” -PP4  Along with maternal and child health matters of the government, these things should also be their priority program, but due to the huge level of migration, the population growth rate has drastically decreased reaching below 1 (. um...), so it is difficult to explain to the local level policymakers that some support should be given to family planning. This data gave false messages to the policymakers, and they have perceived that family planning is no longer required due to population decline.” -PP5  “We had developed integrated family planning service centre in 21 districts. Under this, long-acting family planning service, training, year-round service, and even for permanent family planning services were delivered. But now, its ownership is lost; it's almost collapsed due to changes in governance.” -PP7  “For that, we must allocate a budget for the batch in whichever facility the training is provided. We support training by coordinating with PHTC [Provincial Health Training Centre], and we support for certification. The health coordinators at the health section of the local government should understand that the National Health Training Centre does not need to approve/provide training. It is their responsibility.” -PP7  “In terms of challenges, there are many system related challenges. Officials may have misunderstandings about their role and the process. As _______ [the government agency] is not the one who allocates budget for training. After federalisation, it is the responsibility of local and provincial government to ensure capacity development of the healthcare workers working in the local facilities.” -PP7  “This is also a huge challenge to understand a clear channel and process!” -PP7  “If we get a team of personnel who can supervise, who can mentor healthcare providers as well as provide periodic feedback to the program and hospital managers, this can be successfully implemented.” -PP12  “Health exists beyond health; it is not something that only we can only manage because there are many projects working on women’s empowerment. They can provide great support and work together with us to create demand and raise awareness. When the client comes to the health facility, then we are doing many things.” -PP12  “We are the federal democratic government. Under this federal government, there are 753 municipalities and 7 provinces with local and provincial governments and all federal, local, and provincial governments are at the same level. All are autonomous, and Nobody needs to listen to anybody else's opinion. What has happened now is that we don't have a chain of command.” -PP12 |
| Cost and funding | “…. _______ [an INGO] provided a lot of support until now, but now it seems that it has diverted a bit, and they have started to focus more on adolescent sexual and reproductive health.” -PP7  “However, if we provide PPIUCD with the hope that this works for 12 years, but if women come to remove it in a month, it is a huge economic loss to the government.” -HCP5  “The clients even walk for two days to come to this health facility, causing them huge costs relating to travel, accommodation, cost.” -HCP2  “But the main setback is the loss of copper-T thread, and when they don’t feel or see the IUCD thread, the recipients are required to pay around 3-4 thousand for an ultrasound procedure to find if the copper-T was dislodged and for its removal. ……Initially, it seemed free to patients for insertion, but they perceived it as not free when they faced complications  .” -HCP12  “When we send them to the central radiology department for ultrasound, patients have to pay for the ultrasound procedure.” -HCP12  “Family planning overall is a P1 or priority one programme of Nepal. Government has been investing it well, but the integration of postpartum family planning service hasn’t got enough attention.” -PP2  “……but it is true sometimes we fail to provide the instrument, logistics based on real need. People also have vested political interests in such things. Like politically elected administrative authorities sometimes invest on those things, which are not urgent, to show off and be popular.” -PP3  “Now, to say the truth, RH [Reproductive Health] has been a very neglected issue in the recent times after going to the local level government. There is not much focus from the local government.” -PP4  “The other problem is the demand for the service at the community level. We are not putting enough effort into increasing demand generation. A targeted effort should be done at the community level. Demand must come from the people and the community” -PP4  “Practically, more emphasis is placed on the new programs; family planning is an old program. New projects are created for new issues. Since there are currently fewer projects in family planning, the level of implementation and service delivery has been overshadowed in some places.” -PP5  “Earlier, this used to be a pioneer organisation, and there was no crisis of funding in the past, but in recent times, with a weakening economy due to COVID-19 and Ukraine-Russia war issues such as war refugee management, the external funding has decreased drastically. We worry about how to sustain our activities; that's why we are searching for funding at the local level as well. We are focussing on how to ensure continuity of services and tap the potential of the government’s interest in this field to sustain our health services. We are already taking funding from the local level government to some extent. There is no support from the central government now, but lobbying is going on. Due to the cut-down in the funding given by_______ [an INGO], our major donor organisation, the services are hampered, that's why we are lobbying to increase support for the existing services.” -PP5  “After the global gag rule was introduced, our big service supporter __________ [an international organisation] withdrew its hand from funding.” -PP6  “If you look at the budget, there is still a situation where other partners are providing commodities and support to sustain this program. The IUCD gets even less priority than other contraceptives.” -PP7  “I personally feel that funding priority for family planning has been reduced. The family welfare division itself has also taken initiatives to initiate private hospitals in providing family planning services and have been providing training to the staff.” -PP7  “What we are currently focussing on is the management of complications of pregnancies, maternal problems, newborn problems, a lot of other issues but family planning needs also must be given priority. That is being missed now. We should reinvent this.” -PP9  “Family planning has been neglected in Nepal. That should be accepted…………Currently family planning is not prioritised. Although our focus is both maternal health and reproductive health, our system is focussed more on reducing maternal mortality and reaching SDG goals. TFR is getting low, people in policy positions have started questioning why family planning is needed, which I think they have more to understand why investment in family planning should be continued.” -PP9  “Recently, the overall health budget has been reduced by 30-35%, but the budget reduction should not be a problem because only 60% of our health is being spent in each fiscal year. If we analyse the overall scenario, even if we say that the allocated budget is not enough, 40% of the healthcare budget was not spent in the previous years, and we were unable to spend the allocated funds. But in the current budget, there has been a lot of reduction in the health sector, especially there can be a decline in the budget for family planning, and in this critical scenario, I can’t exactly tell you how much will be allocated to maintain family planning logistics.” -PP9  “…….[an international organisation] has already reduced funding for family planning, and they are more into governance. Development partners such as ……..[an international organisation] buy logistics, …[an international organisation] is focusing more on abortion; another one is focussing on medical abortion. The budget has been reduced, but an extra budget is not needed to provide the service. With the existing staff, we can still implement.” -PP9  “What's more, there is a national trend of investing less in the health sector among other ministries. Because of that, even the province level also invests less in the health sector. And at the local level, budget is mostly allocated for building roads and other infrastructures rather than the health sector.” -PP10  “If we review the family planning program now, the demand generation activities that used to be done in the past few years have decreased very much. We haven’t invested in the community awareness programs, healthcare mobilisation for reaching the dropouts, early marriage couples, young couples.” -PP11  “Now, the total budget of the government of Nepal for health is low, and in that budget, investment in family planning is not even 1%. That is why we are advocating for investment in family planning. Family planning is not only about contraceptives. It is about envisioning your family through your planning.” -PP11  “If we talk about post pregnancy family planning service, it is not satisfactory. What I want to say is that the family planning service itself hasn’t got the level of priority that it should. It has not got the level of resources and importance that it requires.” -PP12  “Frankly speaking, financial resources are a big challenge. Our budget for the healthcare sector is cut off by 30% across the board compared to previous years. It may not be enough to provide basic essential commodities this year. Further, the external funds we get for the family planning commodities have decreased dramatically. There will be a budget deficit of around 4 million (NPR) in last year's requirement only for the commodity. Now, what we need is resources to move forward programmatically; we need human resources, what we have is not enough. This is not an excuse; it is a fact.” -PP12 |
| Little appetite/ compulsion/obligation for change | “We don’t have any target such as PPIUCD prevalence rate to achieve” -HCP1  “Women hesitate to go for permanent contraceptives such as Mini-lap. Women usually prefer Norplant and Depo-Provera.” HP1  “In our area, the most preferred contraceptive is Depo-Provera, followed by the implant. Nowadays, we don’t find clients taking OCPs for a long time. Previously, women were also found to be using OCPs for a long time up to 10 years.” -HCP2  “It’s only about 2,3 cases in a year and Implant is popular compared to IUCD in our facility.” -HCP2  “Women in this area prefer Depo-Provera and implants. Only some women use Copper-T.” -HCP3  “But most of the women deny IUCD so we focus on implant as we can’t give pills to a breastfeeding woman. That is why we are focussing on implants.” -HCP3  “We have logistics and services; only the patients are not receptive at this phase. This creates an awkward situation.” -HCP5  “Earlier, an organisation used to follow up and provide feedback. By doing that, we would have known what we had to do and in what way, and we would have known our shortcomings, but now that is not the case. That's why there is almost a discontinuity in that service.” -HCP6  “I personally think it is an effective contraceptive. But it is very difficult to get patients’ acceptance for this contraceptive.” -HCP7  “At the hospital level, we don’t have any review program to compare targets” -HCP7  “If we look at the data from the last two years, PPIUCD insertion is zero. After that, we haven’t also made any efforts to increase the uptake of the PPIUCD.”  “PPIUCD is a part of SBA training that we receive and following that training, there is no supervision, motivation, or perks. We have never been asked how many PPIUCD have we been providing.” -HCP9  “Although we report the use of family planning, but we have never discussed about what should be the minimum number of PPIUCD that should be provided, why it is less utilised or what made the utilisation more in some months and we have never focussed on the particular contraceptive such as PPIUCD only.” -HCP9  “There was no monitoring body as well. And in the absence of the monitoring body, the implementation of PPIUCD decreased day by day, maybe by 10% today, by 10% next day, and now the whole program has reached a non-existent condition.” -HCP12  “From time to time, we used to get a visit from a professor from the UK but not anymore.” -HCP14  “When a project is first embarked, it runs smoothly and is implemented effectively, but to give it continuity, there are many hurdles, barriers, and many interests. Family planning has a big role in maternity service, but it would be more effective if we had a specific position or directions for service providers.” -HCP14  “But we don’t have specific PPIUCD related contraceptive prevalence rate, or national level PPIUCD prevalence rate.” -PP1  “But if we look comparatively specifically at PPIUCD and IUCD, their utilisation is very low. Their demand is low” -PP1  “We have separate indicators for family planning and safe motherhood, but current information system and reporting system don't seem to have indicators to measure how integrated family planning services and maternity care are.” -PP2 |
| Incentive provision | “The incentives are also very minimal for the healthcare providers. They provide Rs 150 to healthcare provider which is very less compared to the effort healthcare provider put in counselling, convincing and performing sterilisation or putting implant.” -HCP2  “We haven’t got any incentives, especially for providing these services. I have heard that we are entitled to incentives of Rs. 50 for each implant or IUCD service, but I haven’t received them yet. We have family planning in charge in the district public health office, but they never give us incentives for reasons that the budget is not adequate.” -HCP4  “Healthcare providers are provided with a cash incentive of about Rs. 100 for providing IUCD service, similarly, for implant insertion Rs. 60.” -HCP4  “In addition, we have no incentives for providing any of the long-acting reversible contraceptives. It would be good if there were incentives because it also provides some motivation to the staff and it seems that motivation is needed……If we had motivational perks side by side, it would motivate staff to a little extent. They would set a target to increase their service coverage. For example, staff would target to increase 12 another day from 10 people today. If there was an incentive, it would have been better.” -HCP6  “I don't know exactly how much the incentive is, but it seems like 50 rupees for providing IUCD and 30 rupees for the implant service. It is good if the incentives increased, but it is our job, we should be doing it despite if we get incentives or not.” -HCP7  “Now, there is just a nominal incentive for providing contraceptives, not sure, but maybe Rs. 50 or Rs 20. This is just a little number in this context when everything is so expensive………However, incentives can also play a small role.” -HCP9  “Providers also wanted some incentives or some kind of rewards. This issue was also raised during the time of programme implementation. Maybe there was some incentive for the head of the departments but it’s not the head that implements the programme. It was resident doctors and nurses. There were no incentives, and in absence of a central monitoring body and monitoring, the implementation level has not shown much interest since the central body stopped monitoring.” -HCP12 |
| **Barrier theme 5: Contextual factors** | |
| Spouse and hygiene considerations, endemic diseases, critical incidents | “Firstly, vaginal discharge is very common in women here even before insertion.” -HCP1  “They usually live in long-distance relationships… their husbands work as migrant worker in India mostly. So, most women deny it because their husbands are not with them.” -HCP1  “Some have accepted family planning measures within 24 hours when we discharge, but some deny it with reasons such as postpartum bleeding, husbands not being together, and wishing to use family planning in the future. Some promise to come back to get contraceptive services when their husbands visit them as the contraceptive measures will be useless without a partner.” -HCP3  “In our context, most husbands work in India or in foreign countries. Even when women deliver a baby, the husbands are not present. Out of 100 pregnant women, less than 50% of husbands attend during the delivery of babies. As husbands live outside the home most of the time, women don’t use contraceptives often.” -HCP4  “It’s a good option, but in this locality/district, there is a problem with hygiene. Women sleep outside in a hut when menstruating, and don’t wear panties and pads, so there is a high source of infection. Due to this, there is rarely a woman who doesn’t suffer from pelvic inflammatory disease.” -HCP4  “And women would have opted to use contraceptives if their husbands were not abroad……that’s why women also don’t like to use contraceptives in my opinion.” -HCP5  “The main thing is that the husband stays abroad for 4-5 years and naturally, that is one of the reasons for this not coming in their preference.” -HCP6  “And about 50% of the clients report that their husbands reside somewhere else due to jobs. We do our best to provide comprehensive counselling, but mostly they don’t want to use it as they reason that their husbands are abroad.” -HCP9  “Seasonal migration is accepted and established nature of our population. A large proportion, especially the male population of reproductive/productive age, leave this place in search of work…usually, there is a trend to go to India, especially Mumbai or Delhi, in search of work. They come back here during the working season, like May-June, when it is plantation season, and during October-November when it is time to collect the crops. Other times, they spend most of their time there in those Indian cities to earn money. Because they have their own work and employment, they go there. When the husband is gone, women stop using the family planning measures, and when the husband comes back, women use it again.” -PP2  “In fact, we also didn’t recommend in the past using IUCD due to the high prevalence of sexually transmitted infections and HIV in this region. As most men were migrant workers in India, the HIV prevalence was very high, and we especially advocated for the use of barrier methods.” -PP2  “Another big barrier is the barrier of time as the office hours we serve and the people working hours also overlap. But it doesn't have to be full all the time. Once in five years for the implant, once in three months for the three-month injection, so it will not be that big a barrier either.” -PP3  “There is not a single NGO working in family planning in our municipality. Until last year, _____ [an INGO] had an organisation called _____ [an NGO] which worked on abortion and IUCD. Not even one now! Now it seems that organisations working in RH are decreasing.” -PP4  “…a chain of command from the province level to the local level doesn’t exist. The local level has its own plans and programs. Even federal and provincial levels don’t really interfere, saying local level is independent government and local level government are given the constitutional right to carry out basic health services independently.” -PP4  “In the urban sector, we have to think a little differently and create a convincing physical infrastructure at the beginning and ensure that at least some of the eight ANC visits are from the gynaecologist, only then will our population come to our clinics.” -PP4  “In addition, it seems that the health system should be strengthened, especially after the federal system, there is no staff position at various levels of health facilities, and although family planning is Nepal’s priority program. Practically, family planning is an old program, more emphasis is placed on the new programs. New projects are created for new issues. Since there are currently fewer projects in family planning, the level of implementation and service delivery has been overshadowed in some places.” -PP5  “The local government, including municipalities and villages, is providing some kind of grant/financial support. Earlier this used to be a pioneer organisation, and there was no crisis of funding, but in recent times, a weakening economy due to COVID-19 and Ukraine-Russia war issues such as war refugee management, the funding has decreased drastically from support organisation and now the existing services. We worry about how to sustain our activities; that's why we are searching for funding at the local level as well.” -PP5  “But the perineal hygiene is poor among women in rural areas and urban slums there. There is a high prevalence of PID [pelvic inflammatory diseases], STI [sexually transmitted infections] discharges in such areas. When there are these things, their health and hygiene should also be considered. There is no menstrual health hygiene.” -PP6  If we start with access, due to our geographical hardship, the people who should get the service of family planning are not getting the service. There are issues related to access to service. Another thing if we look at the supply side, the service is not provided 24/7 -PP7  “What is a big problem now is that so many partners are outside, and many have relationship outside the marriage. Now the society has a lot of such problems [smiles ...] Maybe not very high percentage but anyways spousal separation is very high and is one reason to decreased CPR. women tend not to use contraceptive when they don’t live with their husband and they meet occasionally, they can conceive due to mistiming. It’s all about education.” -PP9  “Another significant challenge that we face is the restructuring of the health system following federalisation. Before restructuring, there used to be institutional family planning/MCH clinics. There were MCH clinics in every district. Now, they are dismantled during restructuring. It is not available anywhere now. We all know what needs to be done, but there are two big problems here, both in terms of financial and human resources. Another big challenge is the issue of accountability. What we have seen in the current programmes is that budget allocation is not enough; if the implementing management doesn’t take the programme seriously or track their progress, they may get away without putting much effort, showing reasons such as lack of time and work overload.” -PP12 |

Note: ANC: Antenatal care; CFIR – Consolidated Framework for Implementation Research; CPR – Contraceptive prevalence rate; IUCD – Intrauterine Contraceptive Device; MCH – Maternal and Child Health; NGO - Non-governmental organisation; OPD – Outpatient department; PPIUCD – Postpartum Intrauterine Contraceptive Device; TDF – Theoretical Domains Framework; USG: Ultrasonography

**Supplementary Table 5 Illustrative quotes for the enabler themes identified.**

| **Subthemes** | **Illustrative quotes** |
| --- | --- |
| **Enabler theme 1: Reducing unmet need for contraception** | |
| Suboptimal use of contraceptives | “We get many abortion cases; maybe some don’t know they need to use contraceptives after 42 days postpartum, maybe some think they are breastfeeding and don’t need contraceptives, or maybe they don’t want to consult us at all.” -HCP4  “Today, I met a 49-year-old pregnant lady with a 16-year-old son. She still didn't know that she should use such a device because she never had an unplanned pregnancy before.” -HCP8  “When we take their contraceptive history from them, 90% of the cases report not using any family planning devices.” -HCP9  “We should be more focused on the province 2. I am seeing many women with gravida 5,6,7 on a day-to-day basis, and I feel sad for them. You see those women, they look lean, thin, skinny, almost all women are anaemic here. Every second woman is anaemic and if I perform 10 caesarean sections, almost 6-7 cases need blood transfusion. So, we haven't provided enough services during antenatal visits. They have small birth spacing. Usually, almost all deliveries and caesarean sections are conducted with in one-and-a-half year spacing. So, there is much more to work on, especially in this province 2.” -HCP10  “When I recall my childhood, I recall my parents saying, “marna nasakera bancheko” or literally, “survived escaping deaths”. In the previous days, for various reasons, children were born without planning, whether they wanted or not. It may be due to lack of family planning or lack of information, so they may not be able to give the children the opportunity they wanted to the child born without desire and they must have expressed their frustration and pain through their ranting to children. People of similar circumstances are still here in the urban slum area, and there are also some remote settlements here.” -PP3  “And not being able to use family planning even if you know there is a high unmet need of family planning, and the data also shows the same.” -PP3  “From the perspective of supply and demand and population dynamics, there are about five million people outside the country, either for temporary migration or migrant workers. The majority of them are of reproductive age. However, they might need family planning methods. The norms and values of the society have changed a lot. Previously, it was implied that sexual relations were only limited between husbands and wives and contraceptives were not needed as much, but now that is not the case. There is a need for family planning services even when one of the partners is not around. I am not speaking for all, but there are different circumstances for everyone. And if you are not going to get the service or not having access to family planning, you tend to go through the abortion.” -PP7  “When we talk about the contraceptive prevalence rate, people have started questioning why to invest in family planning as it seems people stopped having children due to reduced fertility rate, contraceptive prevalence rate. But when we critically look at it, family planning is not only for birth control, it is related to other various aspects of women, there issues such as unmet needs, raising the rate of abortion.” -PP10  “My understanding is that although emergency contraception is the need of the day, it should not be used for every sexual act. Perhaps it is not so clear to the younger generation that it should be used only as an emergency method. I think there is not enough focus on adolescents.” -PP9 |
| Unintended pregnancy | “Today, I met a 49-year-old pregnant with a 16-year-old son. She still didn't know that she should use such a device because she never had an unplanned pregnancy before.” -HCP8  “There was a case that we met here. One of the clients came with her mother-in-law for a pregnancy test with a 9-month-old baby, and the test was positive. The mother-in-law was happy that the daughter-in-law conceived as she wanted two sons. The first baby was son born through caesarean section. The mother-in-law also believed the next baby was going to be a son and was very happy and decided to keep the baby. I have come across 1-2 such cases. I also suggested them to continue as the siblings grow together.” -HCP13  “In the postpartum phase, around 6 months after having a baby, there are many cases of unknowingly having a baby. In almost every outpatient department, I find one client that conceives at around 6 months postpartum. There was a case of a caesarean section recently; we sent her home with family planning counselling. Since she was not using anything, she returned with pregnancy during her postpartum phase, and we had to carry out the abortion.” -HCP8  “Obviously, fertility can return anytime, and when they don’t use any family planning devices, they go through unplanned pregnancy” -HCP8  “Women being pregnant even in the postpartum period is a common occurrence. I have seen women come with pregnancy after 6-months post caesarean section. This is common, and It’s not a big deal.” -HCP14  “I am prioritising family planning very much because the accidental pregnancy rate of people has increased so much that life has become threatened.” -HCP8  “We have more than 10 abortion cases every month. Sometimes, due to less spacing, they have young children of about 5 months and they experience unwanted pregnancy as they don’t have timing due to their husbands being away and may have unintentional conception whenever they visit their wives. Now, the cases are decreasing. We are giving them post-abortion contraceptives compulsorily, so the number is decreasing. We have really focused on the abortion cases. We are having trouble as we have a low number of staff.” -HCP3  “If we provide this service to many patients, they will be saved from accidental pregnancy, and that means the government's cost will also be saved.” -HCP8  “If PPIUCD is used, it will benefit the whole nation, including the patient parties and healthcare providers, because there will be no unwanted pregnancies as this contraceptive works for 12 years.” -HCP7  “We get many abortion cases; maybe some don’t know they need to use contraceptives after 42 days postpartum, maybe some think they are breastfeeding and don’t need contraceptives, or maybe they don’t want to consult us at all. We have got abortions during the late postpartum phase. They come for abortion with 3 months pregnancy with 5 months old baby in their arm.” -HCP4  “Unwanted pregnancy is inevitable if they don’t use contraceptives. Unwanted pregnancy happens if family planning is not used. After having an unwanted pregnancy, pregnant women should face all negative consequences, that's why we counsel them not to use abortion as a family planning method, but they refrain from using contraceptives due to fear of their side effects. they don't think it's a big deal when they have an abortion, but they think it's a big deal to do family planning. Now that's the situation in Nepal.” -HCP9 |
| High use of abortion for unwanted pregnancy | “The ones who use long-term contraceptives or permanent sterilisation currently, they opt for these options mainly because they already have had 2, 3 abortions.” -HCP2  “The abortion cases are rising; we receive many abortion cases and women have many children; hence, we really emphasise family planning. Women give birth to many children even if they struggle to raise them, and the abortion cases are rising.” -HCP3  “We have more than 10 abortion cases every month. Sometimes, due to less spacing, they have young children of about 5 months and they experience unwanted pregnancy as they don’t have timing due to their husbands being away and may have unintentional conception whenever they visit their wives.” -HCP3  “We have got abortions during the late postpartum phase. They come for abortion with 3 months pregnancy while carrying a 5-month-old baby in their arm.” -HCP4  “Abortion is common even among educated women as they procrastinate to access contraceptives due to busy schedules, maybe! In the last 3-4 years, number of abortions has been very high. Last year, the total number of cases was 350. There were 15 cases in the last month; sometimes it can even go to 20 in a month. The least in 10 in a month. That too from nearby areas of the district headquarters. If all the women would come from all the places of this district, the number would be very high.” -HCP4  “There are a lot of postpartum abortion cases, the child is young, and the pregnancy is aborted, and the abortion is incomplete. A lot of women come here in the emergency with incomplete abortion and bleeding during the late postpartum phase.” -HCP6  “There are also many cases of abortion in the postpartum phase. Mothers who have recently undergone caesarean 4-5 months also come for medical abortion; I remember people who have recently undergone caesarean also come for medical abortion only after 3 months.” -HCP7  “During COVID-19, there were so many CAC [comprehensive abortion care], PAC [post abortion care] and after so many abortions.” -HCP7  “In the postpartum phase, around 6 months after having a baby, there are many cases of unknowingly having a baby. In almost every outpatient department, I find one client that conceives at around 6 months postpartum. There was a case of a caesarean section recently; we sent her home with family planning counselling. Since she was not using anything, she returned with pregnancy during her postpartum phase, and we had to carry out the abortion.” -HCP8  “In many cases, women are taking medicine for abortion directly from outside (meant pharmacies). Consequently, lives are being lost due to medical abortion, and cases of blood transfusion are on the rise.” -HCP8  “There are also some occurrences of postpartum abortions. Abortions that occur within one year of giving birth are few, but it is not unusual.” -HCP9  “…. What can I say if women take abortion pills like popcorns (…shows a concern in the face). The government established reproductive health rights, but how far this is going in the right direction, I am not sure. It's not helping women's rights. It’s increasing the ignorance. The knowledge, attitude and practice of contraception should have increased, but due to the over-the-counter use of medical abortion, it deteriorated further. The incidence of incomplete abortion is increasing.” -HCP10  “There are many postpartum abortion cases. About 10-20% of pregnancies are like that, and abortion is very common. Currently, not many people come directly to the health facilities; instead, they take the abortion pills from the pharmacy for medical abortion. When we ask them if they had an abortion, they say, 'no.' However, when we phrase it as "have you expelled pregnancy with pills?" then they acknowledge that they have taken pills for an abortion. Or We will know later if there are any medical complications. Only a few people come to the hospital; the medicine for medical abortion is like buying vegetables in the market. You can easily buy from anywhere.” -HCP11  “There is a misuse of abortion medicine, especially since the government has given training to pharmacies on how to sell medicine, but they perceive it as permission to distribute the abortion pills. They sell this medicine at a higher price, 4-5 times the original price. And pharmacies ask them to visit hospital if there are any complications. It is a fair deal for them as they don’t have to deal with complications.” -HCP11  “Currently, private drug stores are misusing the law and providing MA [Medical abortion] drugs illegally; unsafe abortion is still hidden due to authorities’ inability to regulate abortion laws and practices. We consider it is as a new challenge.” -PP5  “There are women who come for abortion up to 7 times. Women have misused the abortion, that means abortion seven times.” -PP6 |
| **Enabler theme 2: Integrated PPIUCD Service is beneficial** | |
| Appropriate reproductive behaviour  Perceived benefit to multiple stakeholders  Promotion of women’s autonomy and wellbeing, and neonatal wellbeing | They will also know what happens when babies are born in short intervals. The wellbeing of their babies is also at stake when babies are born in short intervals; mother’s health may deteriorate when babies are born yearly.” -HCP4  “They have small birth spacing. Usually, almost all deliveries and caesarean section are conducted within one-and- a-half year spacing. So, there is much more to work on, especially in this province 2.” -HCP10  “Overall clients were benefitted; this ensures optimal birth spacing.” -HCP12  “If women become pregnant immediately after a caesarean section and they seek abortion, abortion may not be an option particularly in the second and third trimester. However, short birth spacing can increase the risk of uterine rupture, which can be dangerous and contribute to maternal deaths.” -HCP12  “Mainly women are benefitted, but at the same time the government is also indirectly benefitted because the government have invested a lot in health but haven’t got much in return.” -HCP2  “Women are most benefitted due to integration of this service.” -HCP3  “The couple would benefit a lot.” -HCP6  “If PPIUCD is used, it will benefit the whole nation, including the patient parties and healthcare providers. Everyone benefits with family planning coverage.” -HCP7  “If we provide this service to many patients, they will be saved from accidental pregnancy, and that means the government's cost will also be saved.” -HCP8  “Overall clients were benefitted; this provides birth spacing……… Now the other thing is that it helps in decreasing fertility rate, which is desirable at the national level now.” -HCP12  “Major beneficiaries would be the ones who accept and use PPIUCD but that thing directly, indirectly affects the whole family, community as well as the health system, us!” -HCP14  “They will not have lots of children, don’t have to go for abortion, they will be healthy and invest in children’s education and wellbeing.” -HCP3  “Primiparous women are the number one beneficiary from the integrated family planning services because they can get help in deciding what difference they can have babies, how long they can breastfeed.” -HCP4  “I want to give an example of myself; I was young girl when I had my first baby. But I didn’t give birth to a second baby for 8 years. And during that duration, I studied nursing diploma, intermediate, prepared for public service commission exams, and passed them and progressed in my career. When I got information about the family planning, I used it. And with personal experiences too, I know it is important.” -HCP4  “If we manage a little time and provide contraceptive services and counselling, and if clients receive it, I think we can save a lot of lives. Unwanted pregnancy and induced abortion may be the contributing factors for high maternal mortality in our country.” -HCP8  “Family planning helps to reduce maternal mortality. That is why family planning education should be focused.” -HCP8  “Similarly, if there is proper birth spacing, the probability of pre-eclampsia and low birth weight also decreases in the subsequent birth. The babies born will get proper care, and children won’t suffer from malnutrition. Most of the mothers in Nepal come for health check-ups during childbirth only and the hospital delivery rate is also less. However, there has been some increase now. If we give them the choice of contraceptives, we can increase the acceptance of contraceptives and increase the contraceptive prevalence. This will improve the national indicators.” -HCP12  “The abortion rate has decreased. Similarly, there is a decrease in the complications from abortion, bed occupancy may have decreased, and out of pocket expenditure and government expenditure may have decreased. In that way, this directly benefits women and their family and indirectly hospital, community, government.” -HCP14  “Postpartum mothers do not need to return for the insertion of a copper T or any other contraceptive method; it is placed during delivery. This contraceptive option is accessible and convenient for postpartum women.” -HCP12  “Mainly, it benefits both parties. Users have a benefit to themselves. Another perspective is cost-benefit; if we look at it from the government's investment, then the government has many benefits, which means it benefits the whole country. The government can provide low-cost family planning services.” -PP7  “TFR is getting low, and people in policy positions have started questioning why family planning is needed. I think they have more to understand why investment in family planning should be continued. Family planning service is needed for spacing as well.” -PP9 |
| Relative advantage of PPIUCD | “It is a simple process that is less likely to fail. It can be used even if you have other medical conditions. You must wait for some time to use contraceptive pills during breastfeeding time……………its safe compared to other contraceptives.” -HCP1  “Compared to other methods, it’s a long-term temporary method, effective, and has a low failure rate.” -HCP2  “PPIUCD is one of the best non-hormonal methods. Implants, Depo-Provera have many side effects of bleeding, obesity (relating to hormonal effects). PPIUCD is good as it doesn’t have hormonal side effects. But it’s hard to make clients understand.” -HCP3  “If women can ensure its placement and look after it, it is a wonderful option as it doesn’t have any extra medicine; it is only the effect of copper that prevents women from becoming pregnant.” -HCP4  “The side effect is not too much; even if we keep it, it is easy for us. It is not that difficult for patients. It seems to be good compared to other methods during the postpartum phase………PPIUCD seems to be quite effective and It's good too.” -HCP6  “In my opinion, compared to other contraceptives, PPIUCD is good because it works for up to 12 years once you keep it. It is safe. I think it is a good option in terms of health effects if it suits you as this doesn’t contain any hormones.” -HCP7  “However, certain number of people with 2-3 children choose long-term contraceptives as it provides them long-term protection.” -HCP8  “The easiest thing is that it can be kept immediately after childbirth during the postpartum phase. It doesn’t suppress breast milk production as well, and the patient does not need to wait for menstruation to use family planning.” -HCP9  “As a gynaecologist, in my opinion, it has very less side effects, is very effective. Failure rate is very less with IUCD. It is not hormonal, doesn’t include progesterone, or oestrogen (referring to non-hormonal IUCD - commonly practiced in Nepal). Due to which, there are very few side effects.” -HCP10  “I prefer this contraceptive more than other types of contraceptives. If they use pills, they can also miss taking them affecting its effectiveness. Again, you can't give pills until 6 months after the baby is born. There is a possibility of thromboembolism, deep vein thrombosis. There are also some misconceptions about Depo-Provera, pills that they can reduce the breastmilk secretion. This is why we prefer PPIUCD.” -HCP11  “It does not affect the menstrual cycle like other hormonal contraceptives, and it does not need to be taken daily like oral contraceptive pills methods anymore; some people do not use OCPs [oral contraceptive pills], this is hormonal contraceptives due to concerns of their body image. Users complain it spoils the facial image. They also complain of no menstrual cycles, irregular periods, amenorrhoea after taking other long-acting reversible contraceptives even after the removal.” -HCP12  “The failure rate is also low whereas the failure rate of barriers method is high. Although it doesn’t work as permanent family planning, it works as long-term contraceptives…. Because this is not a permanent family planning method, if you want to plan the next baby, it can be taken out anytime and plan again” -HCP12  “I personally think it is a good contraceptive device as this is non-hormonal. it works temporarily, the side-effects are also less, if you want to have a baby, it is easy to take out, no incision is required, easy to remove, easy to palpate where it is.” -HCP13  “If we can explain that it is non-hormonal while other are hormonal, and if the service is delivered with easy access, the uptake could increase.” -PP1  “It is a long-acting contraceptive, non-hormonal. if the context allows, it could be one good option for postpartum mothers.” -PP2  “I feel that even though IUCD is a good contraceptive, it has been overshadowed.” -PP5  “IUCD works for long-term duration, it is non-hormonal, the hormone won’t interfere the function of our body.” -PP7  “It works for long-term which is also beneficial.” -PP8  “Cost-wise, it is more cost-effective than other contraceptives. You can insert after the caesarean section, within 48 hours, even within 6 weeks; it has many advantages. However, its uptake is not as much as we expected.” -PP9  “When we compare this with other options, its non-hormonal, while many other options are hormonal. With this, the side effects caused by hormones are prevented. Other hormonal options have short-term effects compared to this one. It is easier if you want to take it out or discontinue it. Because of that, I think it is better.” -PP10  “PPIUCD is a product brought by extensive scientific research. For those who use it, those who insert it well are doing well. Works for up to 12 years, and is non-hormonal. Since menstruation does not matter, it is one of the best long-acting contraceptives. It doesn't suit some patients, sometimes there can be expulsion on its own, but as a device, it’s the best one.” -PP11 |
| Opportunity during postpartum period | “The IUCD is kept through per vagina. This also carries advantages as sexual diseases can be identified, polyps, erosions, treatment can be done.” -HCP9  “PPIUCD is a very suitable method for the postpartum period because at that moment health care providers get the opportunity to assess the cervix, uterus; women don’t need to worry regarding privacy again, the doctors needn’t assess the depth and size of the uterus again” -HCP4  “The biggest advantage of PPIUCD is that the women of reproductive age come in contact with the hospital during hospital delivery, so this can increase the contraceptive prevalence rate by increasing acceptance and insertion rate increase.” -HCP12  “Its main drawback is the chance of expulsion. It is less likely to occur when kept immediately. That's why it seems good to keep it immediately.” -HCP1  “Women are more receptive to counselling as they are going through difficult phases of pregnancy” -HCP4  “To use other contraceptives, you have to wait for some time, and some people also hesitate to use temporary contraceptives; some don't use them at all.” -HCP6  “They agree to place the IUCD immediately after the delivery because the baby has just been delivered. They don’t have to worry about their privacy and exposing their private parts when the baby is delivered.” -HCP9  “But the postpartum phase is a very vulnerable period, delivery has just happened, and the females won't be happy to keep any foreign object. On the other hand, we provide IUCD with immediate insertion within 10 minutes, within 48 hours or after 6 weeks, but in 48 hours postpartum, they are happy to go back home with their baby, they don't want to go through per speculum examination again. As a female myself, I won't be happy with that. And if we call them after 6 weeks, they are busy lactating mothers; they are least interested in family planning, they are least concerned about birth spacing, and they are happy with their baby. So, this procedure is a bit infeasible. Women will agree with it if it is provided immediately when the placenta is expelled. Otherwise, it is hard to reach clients in the postpartum phase. This is it’s another drawback.” -HCP10  “Postpartum mothers do not need to return for the insertion of a copper T or any other contraceptive method; it is placed during delivery. This contraceptive option is accessible and convenient for postpartum women.” -HCP12  “In my opinion, it is very important to integrate maternity care and family planning. If family planning is provided along with maternal healthcare, if we start family planning services during antenatal care of MCH [maternal and child health], we expect to get good results…… At that period, the counselling can be effective as women may not want to conceive immediately, they may want to have some spacing.” -PP4  “…. study has shown that it is safe, no separate set-up is required, if a PPIUCD is provided in the maternity care post-delivery in the same set-up, two services will be provided at the same time, so it is a huge advantage cost wise as well.” -PP8  “Even if we only manage to counsel women in the postpartum phase or when they are admitted for childbirth where 20,000 to 22,000 pregnant women come for antenatal care, aren’t we covering a significant portion of women?” -PP9 |
| **Enabler theme 3: Policy and partnership** | |
| Availability of equipment and resources | “It is provided free of cost to clients from the government. And sometimes we underestimate the value of the freebies.” -HCP4  “We have PPIUCD sets; we have all the sets for the family planning services such as insertion, and removal sets for PPIUCD. We have never faced stock problem. Sometimes, patients also come here as their closest healthcare facility doesn’t have stock of equipment.” -HCP3  “PPIUCD is provided free of cost all health services from this hospital are free, and on top of PPIUCD is provided free of cost and we have trained workforce. -HCP3  “We have never been out of stock of IUCD. We can provide as many as clients want. I also prepare IUCD set every time. We need to sterilise the set so I should keep doing even if it is not used.” -HCP4  “We have prepared every infrastructure required for PPIUCD; we have PPIUCD set with the anticipation that women will accept the PPIUCD.” -HCP5  “This is a free service. The contraceptives are provided to our hospital by the Nepal government.” -HCP6  “At the moment, the service is not interrupted due to short of IUCD though such was the case in some instances in the recent past.” -HCP6  “We have IUCD sets. They are not being used and therefore there is no such thing as being out of stock. We sometimes are short of implants sometimes, but we never faced shortage of IUCDs so far in this hospital.” -HCP7  “The hospital had adequate equipment and resources.” -HCP7  “All the resources are available. we have all the family planning commodities in stock because we provide inpatient and outpatient safe abortion services under the maternity service. This is why family planning services are all available and for counselling, there is a counselling room. There are flip charts to educate clients.” -HCP9  “One thing is that their background affects them, luckily, if we consider cost, the government is investing a lot, every contraceptive is free of cost from government hospitals. So, it is affordable.” -HCP10  “We have enough resources. This COPPER-T is provided by Public Health office. Most store our instruments and the Copper-T in the ANC ward and in the operational theatre. If there is any shortage, we immediately demand from the public health office.” -HCP11  This is a free service and there is a follow-up visit as well; the hospital will provide the expenses for that. -HCP11  “The necessary instruments for PPIUCD are obviously there, but almost many staff do not even think that PPIUCD should be provided.” -HCP14  “Our health facilities don't have to buy materials for family planning, it is subsidised 100% by the Nepal government, both central and provincial level of the government provide the family planning commodities.” -PP1  “We provide free basic health services throughout the year and along with this, we also carry out programs approved and directed from the federal government, and we monitor private health facilities and monitor the government health facilities as well.” -PP1  “And another important work that we do is the delivery of important program items such as family planning tools, vaccines, TB, leprosy, malaria, which should be provided free of charge, and medicines that must be provided by the government to the local level.” -PP2  “The people who came to the local level will be connected with the service as it happens, now according to the mechanism of the Nepal government, it is very good in the supply of family planning and vaccination.” -PP3  “Similarly, even for the private health facilities, we provide materials such as family planning materials, and they deliver services for free.” -PP3  “In the case of Kathmandu, only people from the lower economic class visit public health clinics if it is free. And even when they come, they came here because it is free not due to good quality. That is the reason why there is not much effort to improve quality of urban public health facilities.” -PP4  “But since our organisation provides free family planning services, the marginalised population/poor population often comes here. Almost 90% of service users are from marginalised population.” -PP5  “These are basic health service provided free of cost to clients granted by the constitution and law.” -PP9  “Now we are giving free service, we are sending mobile teams to some places, but them not coming to take this service even when it is free means that one is not fully conscious about it, not aware of what will happen after using it, of what to do if something happens.” -PP11 |
| Alignment with reproductive health policies and guidelines and current system | “We provide antenatal check-ups according to WHO guidelines.” -HCP1  “It’s a priority 1 program if you look at the policy document. Since this is a priority program of the government, there is no problem at first glance, but -PP7  “We have national family planning guidelines that supports it.” -HCP2  “Family planning is one of the [P1] priority program of Nepal government just like child vaccination program.” -HCP4  “The hospital does not have its own policy, but we are trained during our training. it is the government's policy to provide contraceptive services to women including PPIUCD, postpartum family planning services.” -HCP7  “The government established reproductive health rights, but how far this is going in right direction, I am not sure.” -HCP10  “It is not that there is a separate training for this, but this skill is taught to those who have received SBA training, even to the level of birthing centre.” -PP8  “Our department places high importance to this contraceptive. We ask them to use Copper-t and talk about its liberal approach as they can take it out anytime they like and suggest them of any advantages and why it is more suitable during postpartum phase compared to other contraceptives” -HCP11  “There are things like safe motherhood care policy, we are also bound by such policy and directed our programs and actions.” -HCP14  “Postpartum family planning, delivery services, cafeteria approach counselling, these are basic things. This is given by the constitution as basic rights as well. It should continue.” -HCP14  “From time to time, we receive requests from the federal government or the state government regarding training or capacity building to send providers for training for capacity building, and we arrange this provision, and in our municipality, there are 8-10 nurses who have received IUCD or implants training.” -PP1  “Family planning overall is a P1 or priority one programme of Nepal. Government has been investing it well, but the integration of postpartum family planning service hasn’t got enough attention.” -PP2  “We can fund the trainings. The training sites have been recognised; we should be able to get training from there.” -PP3  “There is a provision of comprehensive family planning and counselling training package from the national training centre under Ministry of Health and Population. Our long serving staff are trained.” -PP4  “There are regulations under the Nepal Health Service Act; there is already a counsellor post, but there are no any counsellors till date.” -PP6  “The government is planning about the integration through the midwives for all the SRH [Sexual and Reproductive Health] services and the newborn services.” -PP6  “The government plans to train employees and with the current shift towards decentralisation due to federalism, it is easy to organise training of the staff as well. For example, if a health post in Biratnagar wants to provide SBA [skilled birth attendant] training to staff, the local government can fund, forward and organise training. There is no necessary to reach to the central government.” -PP7  “The committee does not have to do anything for PPIUCD because it is already approved and we already have guidelines, documents. What we need now is advocacy and another thing is that while forecasting the commodity, PPIUCD also must be forecasted and follow-up and monitoring should be strengthened at the sites where PPIUCD is being provided.” -PP7  “We have started SBA training in Nepal since 2006 with the aim to integrate maternal care. First there were interval IUCDs and other means of family planning. PPIUCD has also been integrated. It has been incorporated in the SBA training package and while giving SBA training, PPIUCD is also taught as a skill.” -PP8  “If you look at the guidelines and all these medical standards, they are at par with international standards. We recently had postpartum family planning strategy meeting. The stakeholders are aware that there is a need of strengthening postpartum family planning services……. the implementation is a bit tricky at the implementation level.” -PP9  “Like if there are two health facilities in a municipality that need trainings for human resource, you can approve trainings through municipality and the municipality is responsible for coordinating trainings. Or you can also conduct training by requesting the province in which that municipality belongs. In addition, in the current system even the federal level health training centres are providing trainings. As our family welfare division is the one to oversee the implementation of family planning services and if it identifies that some services are not being delivered due to unskilled staff, they have also taken initiatives to train staff. They have also coordinated and organised such trainings.” -PP10  “However, even if it seems that the investment is low in health sector, the trainings can be organised contacting the program division at federal level and we have also run few batches of trainings by asking development partner agency for assistance.” -PP10  “Now if you look at the health post, from HA [health assistants] to AHW [auxiliary health workers], ANM [auxiliary nurse midwives], staff nurse, there are four cadres as our mid-level provider, they all get trainings in family planning, they all get training, but how much services they are providing depends on the provider’s attitude, determination and tendency.” -PP11  “We have made many policies. We must work on implementation, the available policy in family planning is enough if we can implement it effectively. Now what we need is implementers and investment to implement. Implementation and demand generation are needed. We are just piling up the policies. It has been well explained that everyone will be given access to reproductive health services in the Safe Motherhood Act. Now, the national HR strategy has become better, but there is no implementation accordingly; that's why we have enough human resources, training materials in PPIUCD; it’s just the implementation aspect that needs improvement from providers and the demand side. We need to engage the providers and create demand together.” -PP11  “However, there is good task shifting in Nepal, nurse providers are very empowered in Nepal. Nurses have contacts with patients, and they are good counsellors as well.” -PP11  “We are training human resources throughout the year. Our organisation has 8 training centres, from that training centre we have been training service providers from remote health posts to any providers from healthcare facilities throughout the year as per request of the organisations or government of Nepal.” -PP11  “We are providing commodities, budgets, trainings, technical and policy level support to all levels but we are not in a position to dictate the terms.” -PP12 |
| Partnership with major implementing agencies | “The family planning services are also provided by other non-governmental organisations as well such as ____[NGO]in the city area. We are also providing financial support to this organisation on an annual basis. Similarly, we also have collaborative relationship with other organisations like______ [ INGO], which provide family planning services.” -PP1  “After the implementation of the federal system, our main stakeholder is the local level of government because the resources have been allocated at the local level, so we coordinate more at the local level………….When there is a shortage of contraceptives or family planning measures such as condoms, pills, depo, IUCD, implants that we practically provide at our clinic point, government health facilities or health office provide us and vice versa.” -PP5  “Basically, we are one of the partners of the Nepali government. Similarly, if you look at the service delivery provision, private sector is also providing services such as ________ [3 NGOs]. Donor agencies such as ____[INGO] is continuously supplying commodities and is also supporting other parts. _________ [another INGO] also supported the programmes. The government division itself has also taken initiatives to initiate private hospitals in providing family planning services and have been providing training to the staff.” -PP7  “We support for training by coordinating with training centre. The health coordinators at health section at the local government should understand that the National Health Training Centre does not need to approve/provide training. It is their responsibility to organise training as required.” -PP7  “Now, many other organisations are also working on it, like professional, non-governmental organisations.” -PP8  “Currently family planning association of Nepal, MSI [Marie Stopes International], PSI [Population Service International] work a less in this field.” -PP9  “Again, transferring has been so good in our system, nurses are all trained in IUCD and Implants. The doctor does not have to do anything, if a little initiation is taken during the free antenatal time, I think it can move on very well.” -PP9  “Everything must be bought, the government also allocates some budget for purchasing and some partners like UNFPA used to purchase for the government, but now the government also allocates budget for this in their budget.” -PP9  “We have a very good relationship with the government. Family welfare division is committed to supporting us and asking us not to downsize and stop our services as our funding is decreased recently. We support each other for the commodities. We also mobilise our providers by collaborating with the government. Now, our organisations are working well in coordination with the local government.” -PP11  “…There is a good task shifting in Nepal, nurse providers are very empowered in Nepal. They have contacts with patients, and they are good counsellors as well.” -PP11  “Health exists beyond health; it is not something that only we can manage because there are many projects working on women empowerment. they can provide great support and work together with us in creating demand, raising awareness. When the client comes to the health facility, then we are doing many things.” -PP12 |

Note: AHW – Auxiliary Health Workers; ANM - Auxiliary Nurse Midwives; HA – Health Assistants; IUCD – Intrauterine contraceptive devices; NGO – Non-Governmental Organisations; INGO – International Non-Governmental Organisations; PPIUCD – Postpartum Intrauterine Contraceptive Device (PPIUCD); SBA – Skilled Birth Attendant; USG – Ultrasonography; TFR – Total Fertility Rate;
